# Mastering diverse control tasks through world models

**DOI:** 10.1038/s41586-025-08744-2

**Published:** 2025-04-02

**Authors:** Danijar Hafner, Jurgis Pasukonis, Jimmy Ba, Timothy Lillicrap

**Affiliations:** 1 Google DeepMind, San Francisco, CA USA; 2 University of Toronto, Toronto, Ontario Canada

**Keywords:** Computer science, Mathematics and computing

## Abstract

Developing a general algorithm that learns to solve tasks across a wide range of applications has been a fundamental challenge in artificial intelligence. Although current reinforcement-learning algorithms can be readily applied to tasks similar to what they have been developed for, configuring them for new application domains requires substantial human expertise and experimentation^1,2^. Here we present the third generation of Dreamer, a general algorithm that outperforms specialized methods across over 150 diverse tasks, with a single configuration. Dreamer learns a model of the environment and improves its behaviour by imagining future scenarios. Robustness techniques based on normalization, balancing and transformations enable stable learning across domains. Applied out of the box, Dreamer is, to our knowledge, the first algorithm to collect diamonds in *Minecraft* from scratch without human data or curricula. This achievement has been posed as a substantial challenge in artificial intelligence that requires exploring farsighted strategies from pixels and sparse rewards in an open world^3^. Our work allows solving challenging control problems without extensive experimentation, making reinforcement learning broadly applicable.

## Abstract (web summary)

A general reinforcement-learning algorithm, called Dreamer, outperforms specialized expert algorithms across diverse tasks by learning a model of the environment and improving its behaviour by imagining future scenarios.

## Main

Reinforcement learning has enabled computers to solve tasks through interaction, such as surpassing humans in the games of Go and Dota^4,5^. It is also a key component for improving large language models beyond what is demonstrated in their pretraining data^6^. Although the proximal policy optimization (PPO) algorithm^7^ has become a standard algorithm in the field of reinforcement learning, more specialized algorithms are often used to achieve higher performance. These specialized algorithms target the unique challenges posed by different application domains, such as continuous control^8^, discrete actions^9,10^, sparse rewards^11^, image inputs^12^, spatial environments^13^ and board games^14^. However, applying reinforcement-learning algorithms to sufficiently new tasks—such as moving from video games to robotics tasks—requires substantial effort, expertise and computational resources for tweaking the hyperparameters of the algorithm^1^. This brittleness poses a bottleneck in applying reinforcement learning to new problems and also limits the applicability of reinforcement learning to computationally expensive models or tasks where tuning is prohibitive. Creating a general algorithm that learns to master new domains without having to be reconfigured has been a central challenge in artificial intelligence and would open up reinforcement learning to a wide range of practical applications.

**Fig. 1:**
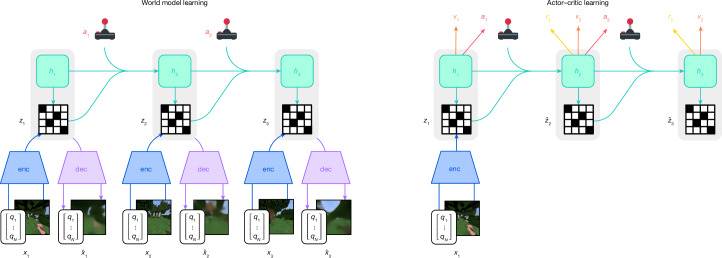
Training process of Dreamer. The world model encodes sensory inputs *x*_*t*_ using the encoder (enc) into discrete representations *z*_*t*_ that are predicted by a sequence model with recurrent state *h*_*t*_ given actions *a*_*t*_. The inputs are reconstructed as $\hat{x}$_*t*_ using the decoder (dec) to shape the representations. The actor and critic predict actions *a*_*t*_ and values *v*_*t*_ and learn from trajectories of abstract representations $\hat{z}$_*t*_ and rewards *r*_*t*_ predicted by the world model.

**Fig. 2:**
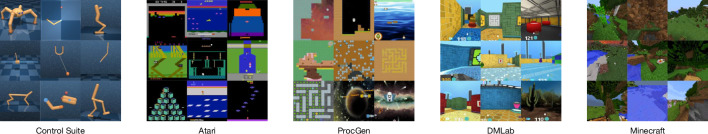
Diverse visual domains used in the experiments. Dreamer succeeds across these domains, ranging from robot locomotion and manipulation tasks over Atari games, procedurally generated ProcGen levels, and DMLab tasks, which require spatial and temporal reasoning, to the complex and infinite world of *Minecraft*. We also evaluate Dreamer on non-visual domains.

Here we present Dreamer, a general algorithm that outperforms specialized expert algorithms across a wide range of domains while using fixed hyperparameters, making reinforcement learning readily applicable to new problems. The algorithm is based on the idea of learning a world model that equips the agent with rich perception and the ability to imagine the future^15–17^. As shown in Fig. 1, the world model predicts the outcomes of potential actions, a critic neural network judges the value of each outcome and an actor neural network chooses actions to reach the best outcomes. Although intuitively appealing, robustly learning and leveraging world models to achieve strong task performance has been an open problem^18^. Dreamer overcomes this challenge through a range of robustness techniques based on normalization, balancing and transformations. We observe robust learning across over 150 tasks from the domains summarized in Fig. 2, as well as across model sizes and training budgets. Notably, larger models not only achieve higher scores but also require less interaction to solve a task, offering practitioners a predictable way to increase performance and data efficiency.

To push the boundaries of reinforcement learning, we consider the popular video game *Minecraft*, which has become a focal point of research in recent years^19–21^, with international competitions held for developing algorithms that autonomously learn to collect diamonds in *Minecraft*^3^. Solving this problem without human data has been widely recognized as a substantial challenge for artificial intelligence because of the sparse rewards, exploration difficulty, long time horizons and the procedural diversity of this open-world game^19^. Owing to these obstacles, previous approaches resorted to using human expert data and domain-specific curricula^20,21^. Applied out of the box, Dreamer is, to our knowledge, the first algorithm to collect diamonds in *Minecraft* from scratch.

## Learning algorithm

We present the third generation of the Dreamer algorithm^22,23^. The algorithm consists of three neural networks: the world model predicts the outcomes of potential actions, the critic judges the value of each outcome, and the actor chooses actions to reach the most valuable outcomes. The components are trained concurrently from replayed experience while the agent interacts with the environment. To succeed across domains, all three components need to accommodate different signal magnitudes and robustly balance terms in their objectives. This is challenging as we are not only targeting similar tasks within the same domain but also aiming to learn across diverse domains with fixed hyperparameters. This section introduces the model components and their robust loss functions.

### World model learning

The world model learns compact representations of sensory inputs through autoencoding^24^ and enables planning by predicting future representations and rewards for potential actions. We implement the world model as a recurrent state-space model^25^, shown in Fig. 1. First, an encoder maps sensory inputs *x*_*t*_ to stochastic representations *z*_*t*_ for each time step *t* in the training sequence. Then, a sequence model with recurrent state *h*_*t*_ predicts the sequence of these representations given past actions *a*_*t*−1_. The concatenation of *h*_*t*_ and *z*_*t*_ forms the model state from which we predict rewards *r*_*t*_ and episode continuation flags *c*_*t*_ ∈ {0, 1} and reconstruct the inputs to ensure informative representations:

$$\begin{array}{lll}\,\mathrm{Sequence\; model:}\, &  & {h}_{t}={f}_{\phi }({h}_{t-1},{z}_{t-1},{a}_{t-1})\\ \,\mathrm{Encoder:}\, &  & {z}_{t} \sim {q}_{\phi }({z}_{t}| {h}_{t},{x}_{t})\\ \,\mathrm{Dynamics\; predictor:}\, &  & {\widehat{z}}_{t} \sim {p}_{\phi }({\widehat{z}}_{t}| {h}_{t})\\ \,\mathrm{Reward\; predictor:}\, &  & {\widehat{r}}_{t} \sim {p}_{\phi }({\widehat{r}}_{t}| {h}_{t},{z}_{t})\\ \,\mathrm{Continue\; predictor:}\, &  & {\widehat{c}}_{t} \sim {p}_{\phi }({\widehat{c}}_{t}| {h}_{t},{z}_{t})\\ \,\mathrm{Decoder:}\, &  & {\widehat{x}}_{t} \sim {p}_{\phi }({\widehat{x}}_{t}| {h}_{t},{z}_{t})\end{array}$$

**Fig. 3:**
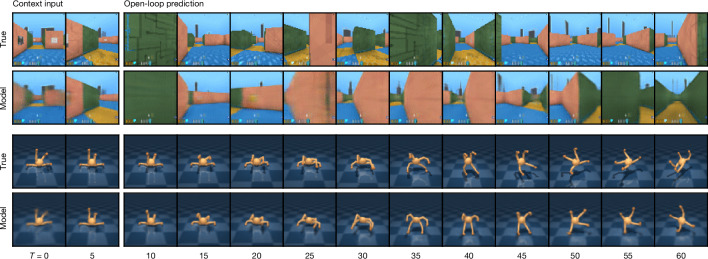
Video predictions of the world model. A procedural maze and a quadrupedal robot are shown. Given 5 context images and the full action sequence of an unseen video, Dreamer predicts 45 frames into the future without access to intermediate images. From pixel observations, the world model learns an understanding of the underlying structure of each environment.

**Extended Data Fig. 1:**
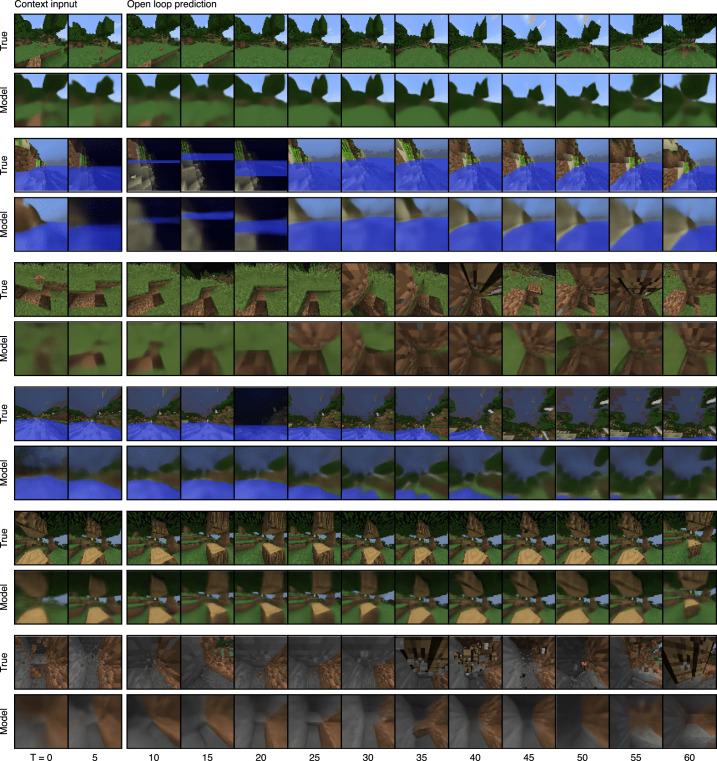
Video predictions of Minecraft. The world model receives the first 5 frames of an unseen video as context input and the predicts 45 steps into the future given the action sequence, without access to intermediate images.

Here, the tilde (~) indicates random variables sampled from their corresponding distribution. Figure 3 visualizes long-term video predictions of the world model. Additional video predictions are shown in Extended Data Fig. 1. The encoder and decoder use convolutional neural networks for image inputs and multilayer perceptrons (MLPs) for vector inputs. The dynamics, reward and continue predictors are also MLPs. The representations are sampled from a vector of softmax distributions and we take straight-through gradients through the sampling step^23^. Given a sequence batch of length *T* with inputs *x*_1:*T*_, actions *a*_1:*T*_, rewards *r*_1:*T*_ and continuation flags *c*_1:*T*_, the world model parameters *ϕ* are optimized end-to-end to minimize the prediction loss ${{\mathcal{L}}}_{{\rm{pred}}}$, the dynamics loss ${{\mathcal{L}}}_{{\rm{dyn}}}$ and the representation loss ${{\mathcal{L}}}_{{\rm{rep}}}$ with corresponding loss weights *β*_pred_ = 1, *β*_dyn_ = 1 and *β*_rep_ = 0.1:

$${\mathcal{L}}(\phi )\doteq {E}_{{q}_{\phi }}[\mathop{\sum }\limits_{t=1}^{T}({\beta }_{{\rm{p}}{\rm{r}}{\rm{e}}{\rm{d}}}{{\mathcal{L}}}_{{\rm{p}}{\rm{r}}{\rm{e}}{\rm{d}}}(\phi )+{\beta }_{{\rm{d}}{\rm{y}}{\rm{n}}}{{\mathcal{L}}}_{{\rm{d}}{\rm{y}}{\rm{n}}}(\phi )+{\beta }_{{\rm{r}}{\rm{e}}{\rm{p}}}{{\mathcal{L}}}_{{\rm{r}}{\rm{e}}{\rm{p}}}(\phi ))],$$

where ${E}_{{q}_{\phi }}$ is the expected value.

The prediction loss trains the decoder and reward predictor via the symlog squared loss described later, and the continue predictor via logistic regression. The dynamics loss trains the sequence model to predict the next representation by minimizing the Kullback–Leibler (KL) divergence between the predictor *p*_*ϕ*_(*z*_*t*_∣*h*_*t*_) and the next stochastic representation *q*_*ϕ*_(*z*_*t*_∣*h*_*t*_, *x*_*t*_). The representation loss, in turn, trains the representations to become more predictable, allowing us to use a factorized dynamics predictor for fast sampling during imagination training. The two losses differ in the stop-gradient operator sg(⋅) and their loss scale. To avoid a degenerate solution where the dynamics are trivial to predict but contain no information about the input, we employ free bits^26^ by clipping the dynamics and representation losses below the value of 1 nat ≈ 1.44 bits. This disables them while they are already minimized well to focus learning on the prediction loss:

$$\begin{array}{c}{{\mathcal{L}}}_{{\rm{p}}{\rm{r}}{\rm{e}}{\rm{d}}}(\phi )\doteq -\log \,{p}_{\phi }({x}_{t}|{z}_{t},{h}_{t})-\log \,{p}_{\phi }({r}_{t}|{z}_{t},{h}_{t})-\log \,{p}_{\phi }({c}_{t}|{z}_{t},{h}_{t})\\ \,{{\mathcal{L}}}_{{\rm{d}}{\rm{y}}{\rm{n}}}(\phi )\doteq \text{max}(1,{\rm{K}}{\rm{L}}[{\rm{s}}{\rm{g}}({q}_{\phi }({z}_{t}|{h}_{t},{x}_{t}))\parallel {p}_{\phi }({z}_{t}|{h}_{t})])\\ \,{{\mathcal{L}}}_{{\rm{r}}{\rm{e}}{\rm{p}}}(\phi )\doteq \text{max}(1,{\rm{K}}{\rm{L}}[{q}_{\phi }({z}_{t}|{h}_{t},{x}_{t})\parallel {\rm{s}}{\rm{g}}({p}_{\phi }({z}_{t}|{h}_{t}))])\end{array}$$

Previous world models require scaling the representation loss differently based on the visual complexity of the environment^22^. Complex scenes contain details unnecessary for control and thus prompt a stronger regularizer to simplify the representations and make them easier to predict. Simple graphics where individual pixels matter for the task require a weaker regularizer to extract fine details. We find that combining free bits with a small representation loss scale resolves this dilemma, allowing for fixed hyperparameters across domains. Moreover, transforming vector observations using the symlog function described later prevents large inputs and large reconstruction gradients, further stabilizing the trade-off with the representation loss.

We occasionally observed spikes in the Kullback–Leibler losses in earlier experiments, consistent with reports for deep variational autoencoders^27^. To prevent this, we parameterize the categorical distributions of the encoder and dynamics predictor as mixtures of 1% uniform and 99% neural network output, making it impossible for them to become deterministic and thus ensuring well-behaved Kullback–Leibler losses. Further model details and hyperparameters are included in Supplementary Information.

### Critic learning

The actor and critic neural networks learn behaviours purely from abstract trajectories of representations predicted by the world model^15^. For environment interaction, we select actions by sampling from the actor network without lookahead. The actor and critic operate on model states *s*_*t*_ ≐ {*h*_*t*_, *z*_*t*_} and thus benefit from the Markovian representations *h*_*t*_ learned by the recurrent world model. The actor aims to maximize the return ${R}_{t}\doteq {\sum }_{\tau =0}^{\infty }{\gamma }^{\tau }{r}_{t+\tau }$ with a discount factor *γ* = 0.997 for each model state, where *τ* denotes imagined future time steps. To consider rewards beyond the prediction horizon *T* = 16, the critic learns to approximate the distribution of returns^28^ for each state under the current actor behaviour:

$${\rm{A}}{\rm{c}}{\rm{t}}{\rm{o}}{\rm{r}}:\,\,\,\,{a}_{t}\sim {\pi }_{\theta }({a}_{t}|{s}_{t})\,\,\,\,\,\,\,\,\text{Critic:}\,\,\,\,{v}_{\psi }({R}_{t}|{s}_{t})$$

Here, *θ* and *ψ* are the parameters of the actor and critic neural networks, respectively. Starting from representations of replayed inputs, the world model and actor generate a trajectory of imagined model states *s*_1:*T*_, actions *a*_1:*T*_, rewards *r*_1:*T*_ and continuation flags *c*_1:*T*_. Because the critic predicts a distribution, we read out its predicted values ${v}_{t}\doteq {\rm{E}}[{v}_{\psi }(\,\cdot \,|{s}_{t})]$ as the expectation of the distribution. To estimate returns that consider rewards beyond the prediction horizon, we compute bootstrapped *λ* returns that integrate the predicted rewards and the values. The critic learns to predict the distribution of the return estimates ${R}_{t}^{\lambda }$ using the maximum likelihood loss:

$$\begin{array}{c}{\mathcal{L}}(\psi )\doteq -\mathop{\sum }\limits_{t=1}^{T}\text{ln}\,{p}_{\psi }({R}_{t}^{\lambda }|{s}_{t})\\ \,{R}_{t}^{\lambda }\doteq {r}_{t}+\gamma {c}_{t}((1-\lambda ){v}_{t}+\lambda {R}_{t+1}^{\lambda })\\ \,{R}_{T}^{\lambda }\doteq {v}_{T}\end{array}$$

Although a simple choice would be to parameterize the critic output as a normal distribution, the return distribution can have multiple modes and vary by orders of magnitude across environments. To stabilize and accelerate learning under these conditions, we parameterize the critic output as a categorical distribution with exponentially spaced bins, decoupling the scale of gradients from the prediction targets as described later. To improve value prediction in environments where rewards are challenging to predict, we apply the critic loss both to imagined trajectories with loss scale *β*_val_ = 1 and to trajectories sampled from the replay buffer with a lower loss scale *β*_repval_ = 0.3. The critic replay loss uses the imagination returns ${R}_{t}^{\lambda }$ at the start states of the imagination rollouts as on-policy value annotations for the replay trajectory to then compute *λ* returns over the replay rewards.

Because the critic regresses targets that depend on its own predictions, we stabilize learning by regularizing the critic towards predicting the outputs of an exponentially moving average of its own parameters. This is similar to target networks used previously in reinforcement learning^9^ but allows us to compute returns using the current critic network. We further noticed that the randomly initialized reward predictor and critic networks at the start of training can result in large predicted rewards that can delay the onset of learning. We thus initialize the output weight matrix of the reward predictor and critic to zeros, which alleviates the problem and accelerates early learning.

### Actor learning

The actor learns to choose actions that maximize return while exploring through an entropy regularizer. However, the correct scale for this regularizer depends on both the scale and the frequency of rewards in the environment. Ideally, we would like the agent to explore more if rewards are sparse and exploit more if rewards are dense or nearby. At the same time, the exploration amount should not be influenced by arbitrary scaling of rewards in the environment. This requires normalizing the return scale while preserving information about reward frequency.

To use a fixed entropy scale of *η* = 3 × 10^−4^ across domains, we normalize returns to be approximately contained in the interval [0, 1]. In practice, subtracting an offset from the returns does not change the actor gradient and thus dividing by the range *S* is sufficient. Moreover, to avoid amplifying noise from function approximation under sparse rewards, we scale down only large return magnitudes and leave small returns below the threshold of *L* = 1 untouched. We use the Reinforce estimator^29^ for both discrete and continuous actions, resulting in the surrogate loss function, where H denotes the policy entropy:

$${\mathcal{L}}(\theta )\doteq -\mathop{\sum }\limits_{t=1}^{T}{\rm{s}}{\rm{g}}(({R}_{t}^{\lambda }-{v}_{t})/\text{max}(1,S))\log \,{\pi }_{\theta }({a}_{t}|{s}_{t})+\,\eta {\rm{H}}[{\pi }_{\theta }({a}_{t}|{s}_{t})]$$

The return distribution can be multi-modal and include outliers, especially for randomized environments where some episodes have higher achievable returns than others. Normalizing by the smallest and largest observed returns would then scale returns down too much and may cause suboptimal convergence. To be robust to these outliers, we compute the range from the 5th to the 95th return percentile (Per) over the batch dimension and smooth out the estimate using an exponential moving average (EMA):

$$S\doteq {\rm{EMA}}({\rm{Per}}({R}_{t}^{\lambda },95)-{\rm{Per}}({R}_{t}^{\lambda },5),0.99)$$

Previous work typically normalizes advantages^7^ rather than returns, emphasizing returns and entropy equally regardless of whether significant returns are within reach or not. Scaling up advantages when rewards are sparse can amplify noise that outweighs the entropy regularizer and stagnates exploration. Normalizing rewards or returns by their standard deviation can fail under sparse rewards where the standard deviation is near zero, which overly amplifies rewards and can cause instabilities. Constrained optimization targets a fixed entropy on average across states^30,31^ regardless of achievable returns, which is robust but explores slowly under sparse rewards and converges lower under dense rewards. We did not find stable hyperparameters across domains for these approaches. Return normalization with a denominator limit overcomes these challenges, exploring rapidly under sparse rewards and converging to high performance across diverse domains.

### Robust predictions

Reconstructing inputs and predicting rewards and returns can be challenging because the scales of these signals vary across domains. Predicting large targets using a squared loss can diverge, whereas L1 and Huber losses^9^ stagnate learning and normalization based on running statistics^7^ introduces non-stationarity. We suggest the symlog squared error as a simple solution to this dilemma. For this, a neural network *f*(*x*, *θ*) with inputs *x* and parameters *θ* learns to predict a transformed version of its targets *y*. To read out predictions $\widehat{y}$ of the network, we apply the inverse transformation:

$${\mathcal{L}}(\theta )\doteq \frac{1}{2}{(f(x,\theta )-{\rm{symlog}}(y))}^{2}\qquad \widehat{y}\doteq {\rm{symexp}}\,(f(x,\theta ))$$

Using the logarithm as transformation would not allow us to predict targets that take on negative values. Therefore, we choose a function from the bi-symmetric logarithmic family^32^ that we name symlog as the transformation with the symexp function as its inverse:

$$\begin{array}{c}{\rm{s}}{\rm{y}}{\rm{m}}{\rm{l}}{\rm{o}}{\rm{g}}(x)\doteq {\rm{s}}{\rm{i}}{\rm{g}}{\rm{n}}(x)\log (|x|+1)\\ {\rm{s}}{\rm{y}}{\rm{m}}{\rm{e}}{\rm{x}}{\rm{p}}(x)\doteq {\rm{s}}{\rm{i}}{\rm{g}}{\rm{n}}(x)(\exp (|x|)-1)\end{array}$$

The symlog function compresses the magnitudes of both large positive and negative values. This allows the optimization process to quickly move the network predictions to large values when needed. The symlog function approximates identity around the origin so it does not affect learning of small enough targets. An alternative asymmetric transformation has previously been proposed for critic learning^33^, which we found less effective on average across domains.

For potentially noisy targets, such as rewards or returns, we additionally introduce the symexp two-hot loss. Here, the network outputs the logits for a softmax distribution over exponentially spaced bins *b*_*i*_ ∈ *B*. Predictions are read out as the weighted average of the bin locations weighted by their predicted probabilities. Importantly, the network can output any continuous value in the supported interval because this weighted average can fall between the buckets:

$$\widehat{y}\doteq {\rm{softmax}}{(f(x))}^{T}B\qquad B\doteq {\rm{symexp}}(\left[\begin{array}{ccc}-20 & \ldots  & +20\end{array}\right])$$

The network is trained on two-hot encoded targets^10,28^, a generalization of one-hot encoding to continuous values. The two-hot encoding of a scalar is a vector with ∣*B*∣ entries that are 0 except at the indices *k* and *k* + 1 of the 2 bins closest to the encoded scalar. The 2 entries sum up to 1, with linearly higher weight given to the bin that is closer to the encoded continuous target. The network is trained to minimize the categorical cross entropy loss for classification with soft targets. The loss depends on only the probabilities assigned to the bins and not on the continuous values associated with the bin locations, fully decoupling the gradient magnitude from the signal scale:

$${\mathcal{L}}(\theta )\doteq -{\rm{t}}{\rm{w}}{\rm{o}}{\rm{h}}{\rm{o}}{\rm{t}}{(y)}^{T}\log \,{\rm{s}}{\rm{o}}{\rm{f}}{\rm{t}}{\rm{m}}{\rm{a}}{\rm{x}}(f(x,\theta ))$$

Applying these principles, Dreamer transforms vector observations using the symlog functions, both for the encoder inputs and the decoder targets and employs the synexp two-hot loss for the reward predictor and critic. We find that these techniques enable robust and fast learning across many diverse domains.

## Evaluation

We evaluate the generality of Dreamer across 8 domains—with over 150 tasks—under fixed hyperparameters. We designed the experiments to compare Dreamer with the best methods in the literature, which are often specifically designed and tuned for the benchmark at hand. We further compare with a high-quality implementation of PPO^7^, a standard reinforcement-learning algorithm that is known for its robustness. We run PPO with fixed hyperparameters chosen to maximize performance across domains, which reproduce strong published results of PPO on ProcGen^34^. To push the boundaries of reinforcement learning, we apply Dreamer to the challenging video game *Minecraft*, comparing it to strong previous algorithms. All Dreamer agents are trained on a single Nvidia A100 graphics processing unit (GPU) each, making it reproducible for many research labs. A public implementation of Dreamer that reproduces all results is available.

### Benchmarks

**Fig. 4:**
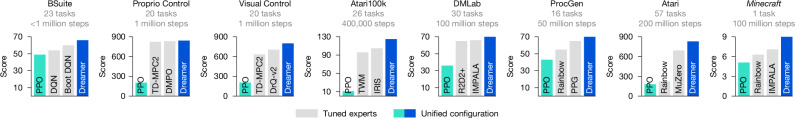
Benchmark scores. Using fixed hyperparameters across all domains, Dreamer outperforms tuned expert algorithms across a wide range of benchmarks and data budgets. Dreamer also substantially outperforms a high-quality implementation of the widely applicable PPO algorithm. IMPALA and R2D2+ use ten times more data on DMLab.

We perform an extensive empirical study across eight domains that include continuous and discrete actions, visual and low-dimensional inputs, dense and sparse rewards, different reward scales, two-dimensional and three-dimensional worlds, and procedural generation. Figure 4 summarizes the benchmark results, comparing Dreamer and a wide range of previous algorithms across diverse domains. Dreamer matches or exceeds the best experts—whether they are model based or model free—in the domains they are applicable to and outperforms PPO across all domains.

- **Atari.** This established benchmark contains 57 Atari 2600 games with a budget of 200 million frames, posing a diverse range of challenges^35^. We use the sticky action simulator setting. Dreamer outperforms the powerful MuZero algorithm^10^ while using only a fraction of the computational resources. Dreamer also outperforms the widely used expert algorithms Rainbow^36^ and IQN^37^.
- **ProcGen.** This benchmark of 16 games features randomized levels and visual distractions to test the robustness and generalization of agents^38^. Within the budget of 50 million frames, Dreamer outperforms the tuned expert algorithms PPG^34^ and Rainbow^38^. Our PPO agent with fixed hyperparameters matches the published score of the highly tuned official PPO implementation^34^.
- **DMLab.** This suite of 30 tasks features three-dimensional environments that test spatial and temporal reasoning^39^. In 100 million frames, Dreamer exceeds the performance of the scalable IMPALA and R2D2+ agents^33^ at 1 billion environment steps, amounting to a data-efficiency gain of over 1,000%. We note that these baselines were not designed for data efficiency but serve as a valuable comparison point for the performance previously achievable at scale.
- **Atari100k.** This data-efficiency benchmark contains 26 Atari games and a budget of only 400,000 frames, amounting to 2 hours of game time^18^. EfficientZero^40^ holds the state of the art by combining online tree search, prioritized replay and hyperparameter scheduling, but also resets levels early to increase data diversity, making a comparison difficult. Without this complexity, Dreamer outperforms the best remaining methods, including the model-based IRIS, TWM and SimPLe agents, and the model-free SPR^41^.
- **Proprio Control Suite.** This benchmark contains 20 simulated robot tasks with continuous actions, proprioceptive vector inputs and a budget of 1 million environment steps^42^. The tasks range from classical control over locomotion to robot manipulation tasks, featuring dense and sparse rewards. Dreamer matches the state of the art on this benchmark, such as DMPO^31^ and TD-MPC2^43^.
- **Visual Control Suite.** This benchmark consists of the same 20 continuous control tasks but the agent receives only high-dimensional images as input^42^. Dreamer establishes a state of the art on this benchmark, outperforming DrQ-v2^44^ and TD-MPC2^43^, which are specialized to visual environments by leveraging data augmentation.
- **BSuite.** This benchmark includes 23 environments with a total of 468 configurations that are specifically designed to test credit assignment, robustness to reward scale and stochasticity, memory, generalization, and exploration^45^. Dreamer establishes a state of the art on this benchmark, outperforming Boot DQN and other methods^46^. Dreamer improves over previous algorithms especially in the scale robustness category.

### *Minecraft*

Collecting diamonds in the popular game *Minecraft* has been a long-standing challenge in artificial intelligence^19–21^. Every episode in this game is set in a unique randomly generated and infinite three-dimensional world. Episodes last until the player dies or up to 36,000 steps equalling 30 minutes, during which the player needs to discover a sequence of 12 items from sparse rewards by foraging for resources and crafting tools. It takes experienced human players about 20 minutes to obtain diamonds^21^. We form a categorical action space of the actions provided by the MineRL competition, which includes abstract crafting actions. Moreover, we follow previous work in accelerating block breaking because learning to hold a button for hundreds of consecutive steps would be infeasible for stochastic policies^20^, allowing us to focus on the essential challenges inherent in *Minecraft*. Refer to Supplementary Information for details and a comparison with video pretraining (VPT) and Voyager^21,47^.

**Fig. 5:**
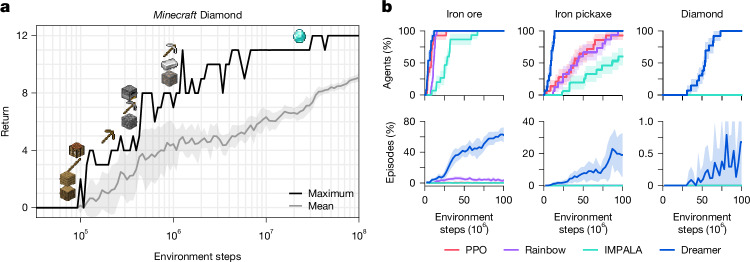
Performance on the *Minecraft* Diamond challenge. **a**, Applied out of the box, Dreamer is, to our knowledge, the first algorithm to accomplish all 12 milestones leading up to the diamond, from sparse rewards without human data or curricula. **b**, Fraction of trained agents that discover each of the three latest items in the diamond task, and the fraction of episodes during which they obtain the item. Although previous algorithms progress up to the iron pickaxe, Dreamer is the only compared algorithm that discovers diamonds, and does so in every training run. Shaded areas indicate one standard deviation.

**Extended Data Fig. 2:**
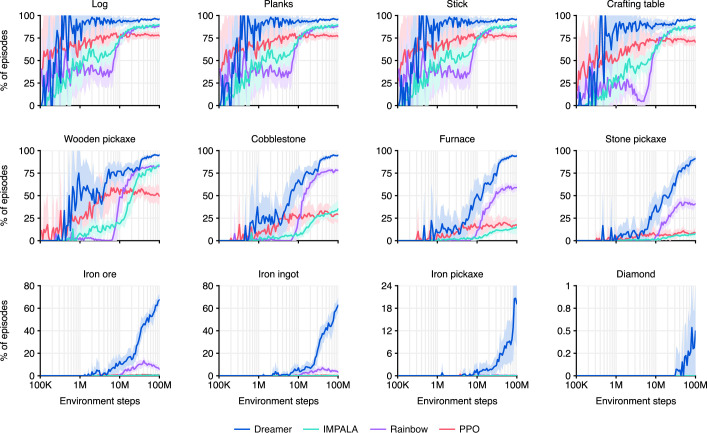
Minecraft item success rates. Dreamer obtains items at substantially higher rates than the baselines and continues to improve on hard items throughout training.

Because of the training time in this complex domain, extensive tuning would be difficult for *Minecraft*. Instead, we apply Dreamer out of the box with its default hyperparameters. As shown in Fig. 5, Dreamer is, to our knowledge, the first algorithm to collect diamonds in *Minecraft* from scratch without using human data, as required by VPT^21^, or adaptive curricula^20^. All the Dreamer agents discover diamonds within 100 million environment steps. Success rates of all items are shown in Extended Data Fig. 2. Although several strong baselines progress to advanced items such as the iron pickaxe, none of them discover a diamond.

### Ablations

**Fig. 6:**
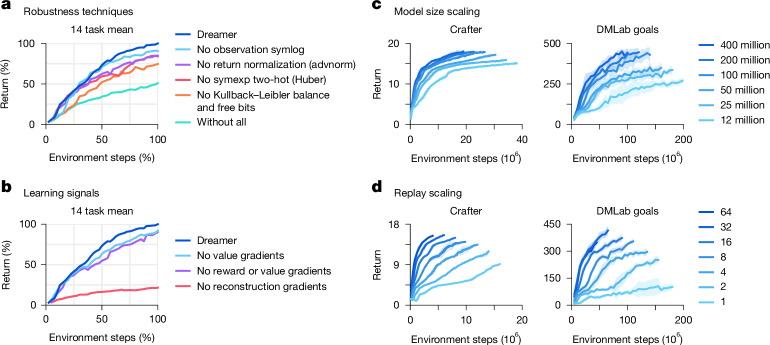
Ablations and robust scaling of Dreamer. **a**, All individual robustness techniques contribute to the performance of Dreamer on average, although each individual technique may affect only some tasks. Training curves of individual tasks are included in Supplementary Information. advnorm, advantage normalization. **b**, The performance of Dreamer predominantly rests on the unsupervised reconstruction loss of its world model, unlike most previous algorithms that rely predominantly on reward and value prediction gradients^7,9,10^. **c**, The performance of Dreamer increases monotonically with larger model sizes, ranging from 12 million to 400 million parameters. Notably, larger models not only increase task performance but also require less environment interaction. **d**, Higher replay ratios predictably increase the performance of Dreamer. Together with model size, this allows practitioners to improve task performance and data efficiency by employing more computational resources.

In Fig. 6, we ablate the robustness techniques and learning signals on a diverse set of 14 tasks to understand their importance. The training curves of individual tasks are included in Supplementary Information. We observe that all robustness techniques contribute to performance, most notably the Kullback–Leibler balancing and free bits of the world model objective, followed by return normalization and symexp two-hot regression for reward and value prediction. In general, we find that each individual technique is critical on a subset of tasks but may not affect performance on other tasks. To investigate the effect of the world model, we ablate the learning signals of Dreamer by stopping either the task-specific reward and value prediction gradients or the task-agnostic reconstruction gradients from shaping its representations. Whereas previous reinforcement-learning algorithms often rely only on task-specific learning signals^9,10^, Dreamer rests predominantly on the unsupervised objective of its world model. This finding could allow for future algorithm variants that leverage pretraining on unsupervised data.

### Scaling properties

To investigate whether Dreamer can scale robustly, we train 6 model sizes ranging from 12 million to 400 million parameters, as well as different replay ratios on Crafter^48^ and a DMLab task^39^. The replay ratio affects the number of gradient updates performed by the agent. Figure 6 shows robust learning with fixed hyperparameters across the compared model sizes and replay ratios. Moreover, increasing the model size directly translates to both higher task performance and a lower data requirement. Increasing the number of gradient steps further reduces the interactions needed to learn successful behaviours. The results show that Dreamer learns robustly across model sizes and replay ratios, providing a predictable way of increasing its performance by scaling computational resources.

## Previous work

Developing general-purpose algorithms has long been a goal of reinforcement-learning research. PPO^7^ is widely used and robust but requires large amounts of experience and often underperforms specialized alternatives. MuZero^10^ plans over discrete actions using a value prediction model, but the authors did not release an implementation and the algorithm contains several complex components, making it challenging to reproduce. Gato^49^ fits one model to expert demonstrations of multiple tasks but cannot improve autonomously. In comparison, Dreamer masters a diverse range of environments with fixed hyperparameters, does not require expert data and its implementation is open source.

*Minecraft* has been a focus of recent research. MineRL offers several competition environments and a diverse human dataset to support exploring and learning skills^19^. VPT^21^ recorded contractor gameplay with keyboard and mouse actions for behaviour cloning followed by reinforcement learning, obtaining diamonds using 720 GPUs for 9 days. Voyager uses a language model to call the commands of the MineFlayer bot scripting layer that was specifically engineered to the game and exposes high-level actions^47^. Dreamer uses the MineRL competition action space that includes abstract crafting actions to autonomously learn to collect diamonds from sparse rewards using 1 GPU for 9 days, without human data.

Learning dynamics models of unknown environments and using them for reinforcement learning^15^ has been explored in early algorithms, such as PILCO, E2C and Visual Foresight^16^. PlaNet introduced a latent dynamics model accurate enough to plan from pixels^25^. IRIS and TWM^41^ integrate transformers, whereas R2I employs structured state-space models for long-term memory^50^. TD-MPC2^43^ learns deterministic dynamics to combine a policy network with classical planning for continuous actions and employs robustness techniques of Dreamer, such as percentile return normalization.

## Conclusion

We present the third generation of the Dreamer algorithm, a general reinforcement-learning algorithm that masters a wide range of domains with fixed hyperparameters. Dreamer not only excels across over 150 tasks but also learns robustly across varying data and compute budgets, moving reinforcement learning towards a wide range of practical applications. Applied out of the box, Dreamer is, to our knowledge, the first algorithm to collect diamonds in *Minecraft* from scratch, achieving a significant milestone in the field of artificial intelligence. As a high-performing algorithm that is based on a learned world model, Dreamer paves the way for future research directions, including teaching agents world knowledge from internet videos and learning a single world model across domains to allow artificial agents to build up increasingly general knowledge and competency.

## Methods

### Baselines

#### PPO

We employ the PPO algorithm^7^, which has become a standard choice in the field, to compare Dreamer under fixed hyperparameters across all benchmarks. There are a large number of PPO implementations available publicly and they are known to substantially vary in task performance^2^. To ensure a comparison that is representative of the highest performance PPO can achieve under fixed hyperparameters across domains, we choose the high-quality PPO implementation available in the Acme framework^51^ and select its hyperparameters in Extended Data Table 6 following recommendations^1,2^ and additionally tune its epoch batch size to be large enough for complex environments^38^, its learning rate and its entropy scale. We match the discount factor to Dreamer because it works well across domains and is a frequent choice in the literature^10,33^. We choose the IMPALA network architecture that we have found performed better than alternatives^38^ and set the minibatch size to the largest possible for one A100 GPU. We verify the performance of our PPO implementation and hyperparameters on the ProcGen benchmark, where a highly tuned PPO implementation has been reported by the PPO authors^34^. We find that our implementation matches or slightly outperforms this performance reference. The training time of the implementation is comparable to Dreamer under equal replay ratio. It runs about 10-times faster than Dreamer with a train ratio of 32, unless restricted by environment speed, owing to its inherently lower experience reuse.

#### Additional baselines

For *Minecraft*, we additionally tune and run the IMPALA and Rainbow algorithms because successful end-to-end learning from scratch has not been reported in the literature^19^. We use the Acme implementations^51^ of these algorithms, use the same IMPALA network we used for PPO, and tune the learning rate and entropy regularizers. For continuous control, we run the official implementation of TD-MPC2^43^ from proprioceptive inputs and from images. We note that the code applies data augmentation and frame stacking for visual inputs—which is not documented in its paper—which is crucial to its performance. The training time of TD-MPC2 is 1.3 days for proprioceptive inputs and 8.0 days from pixels. Besides that, we compare with a wide range of tuned expert algorithms reported in the literature^9,10,33,36,41,44,52–54^.

### Benchmarks

Aggregated scores on all benchmarks are shown in Extended Data Table 1. Scores and training curves of individual tasks are included in Supplementary Information.

#### Protocols

Summarized in Extended Data Table 2, we follow the standard evaluation protocols for the benchmarks where established. Atari^35^ uses 57 tasks with sticky actions^55^. The random and human reference scores used to normalize scores vary across the literature and we chose the most common reference values, replicated in Supplementary Information. DMLab^39^ uses 30 tasks^52^ and we use the corrected action space^33,56^. We evaluate at 100 million steps because running for 10 billion as in some previous work was infeasible. Because existing published baselines perform poorly at 100 million steps, we compare with their performance at 1 billion steps instead, giving them a 10-times data advantage. ProcGen uses the hard difficulty setting and the unlimited level set^38^. Previous work compares at different step budgets^34,38^ and we compare at 50 million steps owing to computational cost, as there is no action repeat. For *Minecraft* Diamond purely from sparse rewards, we establish the evaluation protocol to report the episode return measured at 100 million environment steps, corresponding to about 100 days of in-game time. Atari100k^18^ includes 26 tasks with a budget of 400,000 environment steps, 100,000 after action repeat. Previous work has used various environment settings, summarized in Extended Data Table 3, and we chose the environments as originally introduced. Visual control and proprioceptive control span the same 20 tasks^22,42^ with a 1 million budget. Across all benchmarks, we use no action repeat unless prescribed by the literature.

#### Environment instances

In earlier experiments, we observed that the performance of both Dreamer and PPO is robust to the number of environment instances. On the basis of the central processing unit resources available on our training machines, we use 16 environment instance by default. For BSuite, the benchmark requires using a single environment instance. We also use a single environment instance for Atari100K because the benchmark has a budget of 400,000 environment steps whereas the maximum episode length in Atari is in principle 432,000 environment steps. For *Minecraft*, we use 64 environments using remote central processing unit workers to speed up experiments because the environment is slower to step.

#### Seeds and error bars

We run five seeds for each Dreamer and PPO per benchmark, with the exception of ten seeds for BSuite as required by the benchmark and ten seeds for *Minecraft* to reliably report the fraction of runs that achieve diamonds. All curves show the mean over seeds with one standard deviation shaded.

#### Computational choices

All Dreamer and PPO agents in this paper were trained on a single Nvidia A100 GPU each. Dreamer uses the 200 million model size by default. The replay ratio controls the trade-off between computational cost and data efficiency as analysed in Fig. 6 and is chosen to fit the step budget of each benchmark.

### Previous generations

We present the third generation of the Dreamer line of work. Where the distinction is useful, we refer to this algorithm as DreamerV3. The DreamerV1 algorithm^22^ was limited to continuous control, the DreamerV2 algorithm^23^ surpassed human performance on Atari, and the DreamerV3 algorithm enables out-of-the-box learning across diverse benchmarks.

We summarize the changes introduced for DreamerV3 as follows:

- Robustness techniques: observation symlog, combining Kullback–Leibler balance with free bits, 1% unimix for categoricals in the recurrent state-space model and actor, percentile return normalization, symexp two-hot loss for the reward head and critic
- Network architecture: block gated recurrent unit (block GRU), RMSNorm normalization, sigmoid linear unit (SiLu) activation
- Optimizer: adaptive gradient clipping, LaProp (RMSProp before momentum)
- Replay buffer: larger capacity, online queue, storing and updating latent states.

### Implementation

#### Model sizes

To accommodate different computational budgets and analyse robustness to different model sizes, we define a range of models shown in Extended Data Table 4. The sizes are parameterized by the model dimension, which approximately increases in multiples of 1.5, alternating between power of 2 and power of 2 scaled by 1.5. This yields tensor shapes that are multiples of eight as required for hardware efficiency. Sizes of different network components derive from the model dimension. The MLPs have the model dimension as the number of hidden units. The sequence model has eight times the number of recurrent units, split into eight blocks of the same size as the MLPs. The convolutional encoder and decoder layers closest to the data use 16-times-fewer channels than the model dimension. Each latent also uses 16-times-fewer codes than the model dimension. The number of hidden layers and number of latents is fixed across model sizes. All hyperparamters, including the learning rate and batch size, are fixed across model sizes.

#### Hyperparameters

Extended Data Table 5 shows the hyperparameters of Dreamer. The same setting is used across all benchmarks, including proprioceptive and visual inputs, continuous and discrete actions, and two-dimensional and three-dimensional domains. We do not use any annealing, prioritized replay, weight decay or dropout.

#### Networks

Images are encoded using stride 2 convolutions to resolution 6 × 6 or 4 × 4 and then flattened and decoded using transposed stride 2 convolutions, with sigmoid activation on the output. Vector inputs are symlog transformed and then encoded and decoded using three-layer MLPs. The actor and critic neural networks are also three-layer MLPs and the reward and continue predictors are one-layer MLPs. The sequence model is a GRU^57^ with block-diagonal recurrent weights^58^ of eight blocks to allow for a large number of memory units without quadratic increase in parameters and computation. The input to the GRU at each time step is a linear embedding of the sampled latent *z*_*t*_, of the action *a*_*t*_, and of the recurrent state to allow mixing between blocks.

#### Distributions

The encoder, dynamics predictor and actor distributions are mixtures of 99% of the predicted softmax output and 1% of a uniform distribution^59^ to prevent zero probabilities and infinite log probabilities. The rewards and critic neural networks output a softmax distribution over exponentially spaced bins *b* ∈ *B* and are trained towards two-hot encoded targets:

$${\rm{t}}{\rm{w}}{\rm{o}}{\rm{h}}{\rm{o}}{\rm{t}}{(x)}_{i}\doteq \left\{\begin{array}{cc}|{b}_{k+1}-x|/|{b}_{k+1}-{b}_{k}|\, & {\rm{i}}{\rm{f}}\,i=k\\ |{b}_{k}-x|/|{b}_{k+1}-{b}_{k}|\, & {\rm{i}}{\rm{f}}\,i=k+1\\ 0\, & {\rm{e}}{\rm{l}}{\rm{s}}{\rm{e}}\end{array}\right.\,k\doteq \mathop{\sum }\limits_{j=1}^{|B|}\delta ({b}_{j} < x)$$

In the equation, *δ* refers to the indicator function. The output weights of two-hot distributions are initialized to zero to ensure that the agent does not hallucinate rewards and values at initialization. For computing the expected prediction of the softmax distribution under bins that span many orders of magnitude, the summation order matters, and positive and negative bins should be summed up separately, from small to large bins, and then added. Refer to the source code for an implementation.

#### Optimizer

We employ adaptive gradient clipping^60^, which clips per-tensor gradients if they exceed 30% of the L2 norm of the weight matrix they correspond to, with its default *ϵ* = 10^−3^. Adaptive gradient clipping decouples the clipping threshold from the loss scales, allowing to change loss functions or loss scales without adjusting the clipping threshold. We apply the clipped gradients using the LaProp optimizer^61^ with *ϵ* = 10^−20^ and its default parameters *β*_1_ = 0.9 and *β*_2_ = 0.99. LaProp normalizes gradients by RMSProp and then smoothes them by momentum, instead of computing both momentum and normalizer on raw gradients as Adam does^62^. This simple change allows for a smaller epsilon and avoids occasional instabilities that we observed under Adam.

#### Experience replay

We implement Dreamer using a uniform replay buffer with an online queue^63^. Specifically, each minibatch is formed first from non-overlapping online trajectories and then filled up with uniformly sampled trajectories from the replay buffer. We store latent states into the replay buffer during data collection to initialize the world model on replayed trajectories, and write the fresh latent states of the training rollout back into the buffer. Although prioritized replay^64^ is used by some of the expert algorithms we compare with and we found it to also improve the performance of Dreamer, we opt for uniform replay in our experiments for ease of implementation. We parameterize the amount of training via the replay ratio. This is the fraction of time steps trained on per time step collected from the environment, without action repeat. Dividing the replay ratio by the time steps in a minibatch and by action repeat yields the ratio of gradient steps to environment steps. For example, a replay ratio of 32 on Atari with action repeat of 4 and batch shape 16 × 64 corresponds to 1 gradient step every 128 environment steps, or 1.5 million gradient steps over 200 million environment steps.

### *Minecraft*

#### Game description

With 100 million monthly active users, *Minecraft* is one of the most popular video games worldwide. *Minecraft* features a procedurally generated three-dimensional world of different biomes, including plains, forests, jungles, mountains, deserts, taiga, snowy tundra, ice spikes, swamps, savannahs, badlands, beaches, stone shores, rivers and oceans. The world consists of 1-m-sized blocks that the player can break and place. There are about 30 different creatures that the player can interact with or fight. From gathered resources, the player can use over 350 recipes to craft new items and progress through the technology tree, all while ensuring safety and food supply to survive. There are many conceivable tasks in *Minecraft* and as a first step, the research community has focused on the salient task of obtaining diamonds, a rare item found deep underground and that requires progressing through the technology tree.

#### Learning environment

We built the *Minecraft* Diamond environment on top of MineRL v0.4.4^19^, which offers abstract crafting actions. The *Minecraft* version is 1.11.2. We make the environment publicly available as a faithful version of MineRL that is ready for reinforcement learning with a standardized action space. To make the environment usable for reinforcement learning, we define a flat categorical action space and fix bugs that we discovered with the original environments via human play testing. For example, when breaking diamond ore, the item sometimes jumps into the inventory and sometimes needs to be collected from the ground. The original environment terminates episodes when breaking diamond ore so that many successful episodes end before collecting the item and thus without the reward. We remove this early termination condition and end episodes when the player dies or after 36,000 steps, corresponding to 30 minutes at the control frequency of 20 Hz. Another issue is that the game sometimes misses the jump key when it is pressed and released quickly, which we solve by keeping the key pressed for 200 ms. The camera pitch is limited to a 120° range to avoid singularities.

#### Observations and rewards

The agent observes a 64 × 64 × 3 first-person image, an inventory count vector for the over 400 items, a vector of maximum inventory counts since episode begin to tell the agent which milestones it has achieved, a one-hot vector indicating the equipped item, and scalar inputs for the health, hunger and breath levels. We follow the sparse reward structure of the MineRL competition environment^19^ that rewards 12 milestones leading up to the diamond, for obtaining the items log, plank, stick, crafting table, wooden pickaxe, cobblestone, stone pickaxe, iron ore, furnace, iron ingot, iron pickaxe and diamond. The reward for each item is given only once per episode, and the agent has to learn to collect certain items multiple times to achieve the next milestone. To make the return easy to interpret, we give a reward of +1 for each milestone instead of scaling rewards based on how valuable each item is. In addition, we give −0.01 for each lost heart and 0.01 for each restored heart, but did not investigate whether this is helpful.

#### Action space

Although the MineRL competition environment^19^ is an established standard in the literature^3,20^, it provides a complex dictionary action space that requires additional set-up to connect agents. The action space provides entries for camera movement using the mouse, keyboard keys for movement, mouse buttons for mining and interacting, and abstract inventory actions for crafting and equipping items. To connect the environment to reinforcement-learning agents, we turn them into a categorical space in the simplest possible way, yielding the 25 actions listed in Extended Data Table 7. These map onto keyboard keys, mouse buttons, camera movement and abstract inventory actions. The jump action presses the jump and forward keys, because the categorical action space allows only one action at a time and the jump key alone would only allow jumping in place rather than onto something. A similar but more complex version of this action space was used for curriculum learning in *Minecraft* in the literature^20^.

#### Break speed

In *Minecraft*, breaking blocks requires keeping the left mouse button pressed continuously for a few seconds, corresponding to hundreds of time steps at 20 Hz. For an initially uniform categorical policy with 25 actions, the chance of breaking a wood block that is already in front of the player would thus be ${\frac{1}{25}}^{400}\approx 1{0}^{-560}$. This makes the behaviour impossible to discover from scratch without priors of how humans use computers. Although this challenge could be overcome with specific inductive biases, such as learned action repeat^65^, we argue that learning to keep the same button pressed for hundreds of steps does not lie at the core of what makes *Minecraft* an interesting challenge for artificial intelligence. To allow agents to learn to break blocks, we therefore follow previous work and increase the block-breaking speed^20^, so that blocks break within a few time steps depending on the material. As can be seen from the tuned baselines, the resulting environment still poses a significant challenge to current learning algorithms.

#### Other environments

Voyager uses the substantially more abstract actions provided by the high-level MineFlayer bot scripting library, such as predefined behaviours for exploring the world until a resource is found and for automatically mining specified materials within a 32-m distance^47^. It also uses high-level semantic observations instead of images. Unlike the Voyager environment, the MineRL competition environment requires visual perception and low-level actions for movement and the camera, such as having to jump to climb onto a block or rotate the camera to face a block for mining. VPT^21^ uses mouse movement for crafting and does not speed up block breaking, making it more challenging than the MineRL competition action space but easier to source corresponding human data. To learn under this more challenging set-up, its authors leverage significant domain knowledge to design a hierarchical action space composed of 121 actions for different foveated mouse movements and 4,230 meaningful key combinations. In summary, we recommend the MineRL competition environment with our categorical action space when a simple set-up is preferred, the Voyager action space for prompting language models without perception or low-level control, and the VPT action space when using human data.

## Online content

Any methods, additional references, Nature Portfolio reporting summaries, source data, extended data, supplementary information, acknowledgements, peer review information; details of author contributions and competing interests; and statements of data and code availability are available at 10.1038/s41586-025-08744-2.

## Supplementary information

Supplementary Information Detailed empirical results in the form of learning curve graphs and numeric score tables for all tasks.

## Figures and Tables

**Extended Data Table 1:**
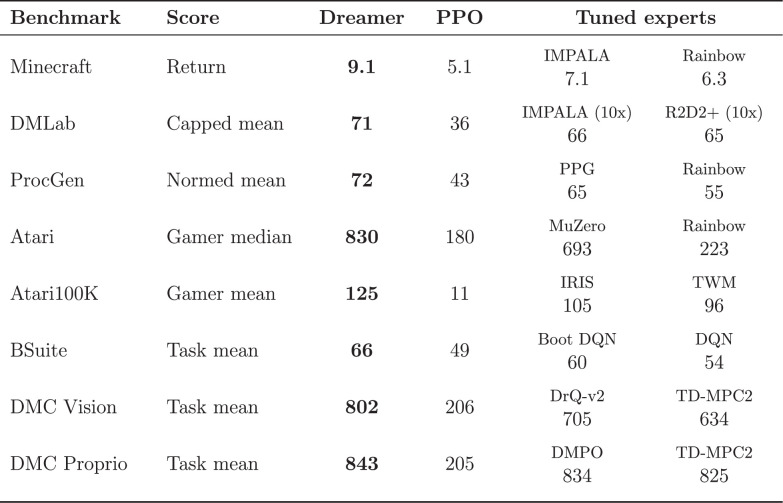
Benchmark scores

**Extended Data Table 2:**
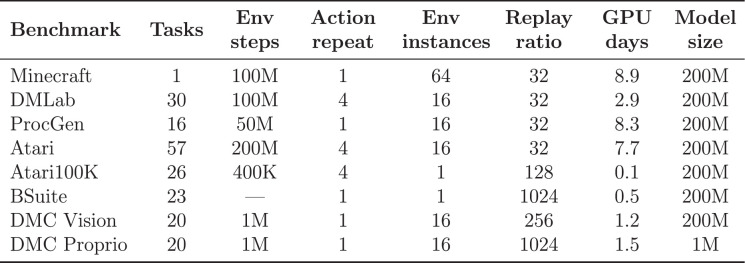
Benchmark overview

**Extended Data Table 3:**
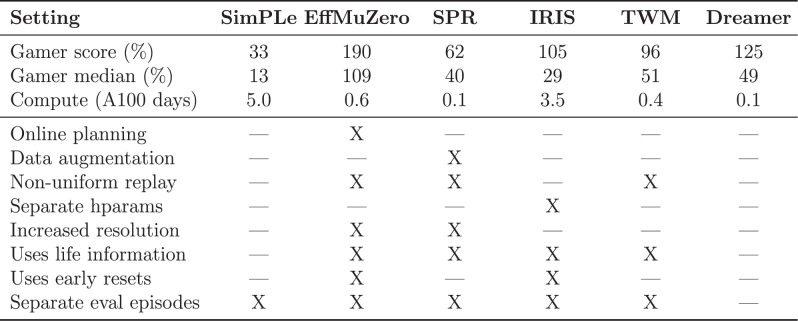
Deviations from the original Atari100k protocol of SimPLe

**Extended Data Table 4:**
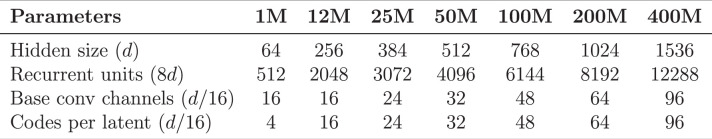
Dreamer model sizes

**Extended Data Table 5:**
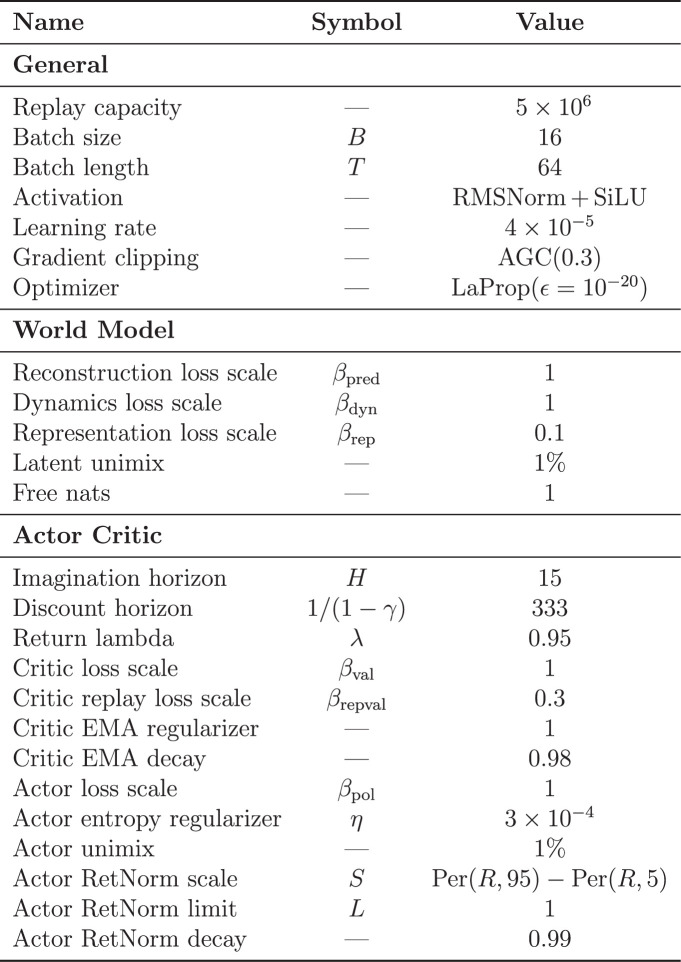
Dreamer hyperparameters

**Extended Data Table 6:**
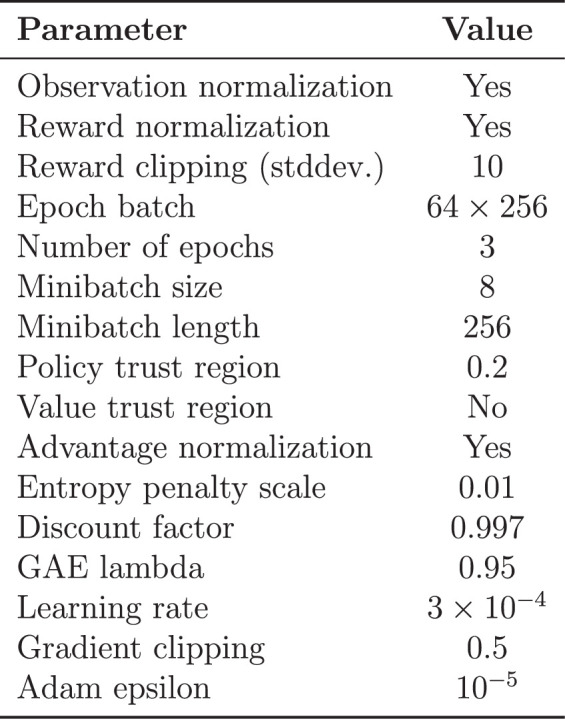
PPO hyperparameters

**Extended Data Table 7:**
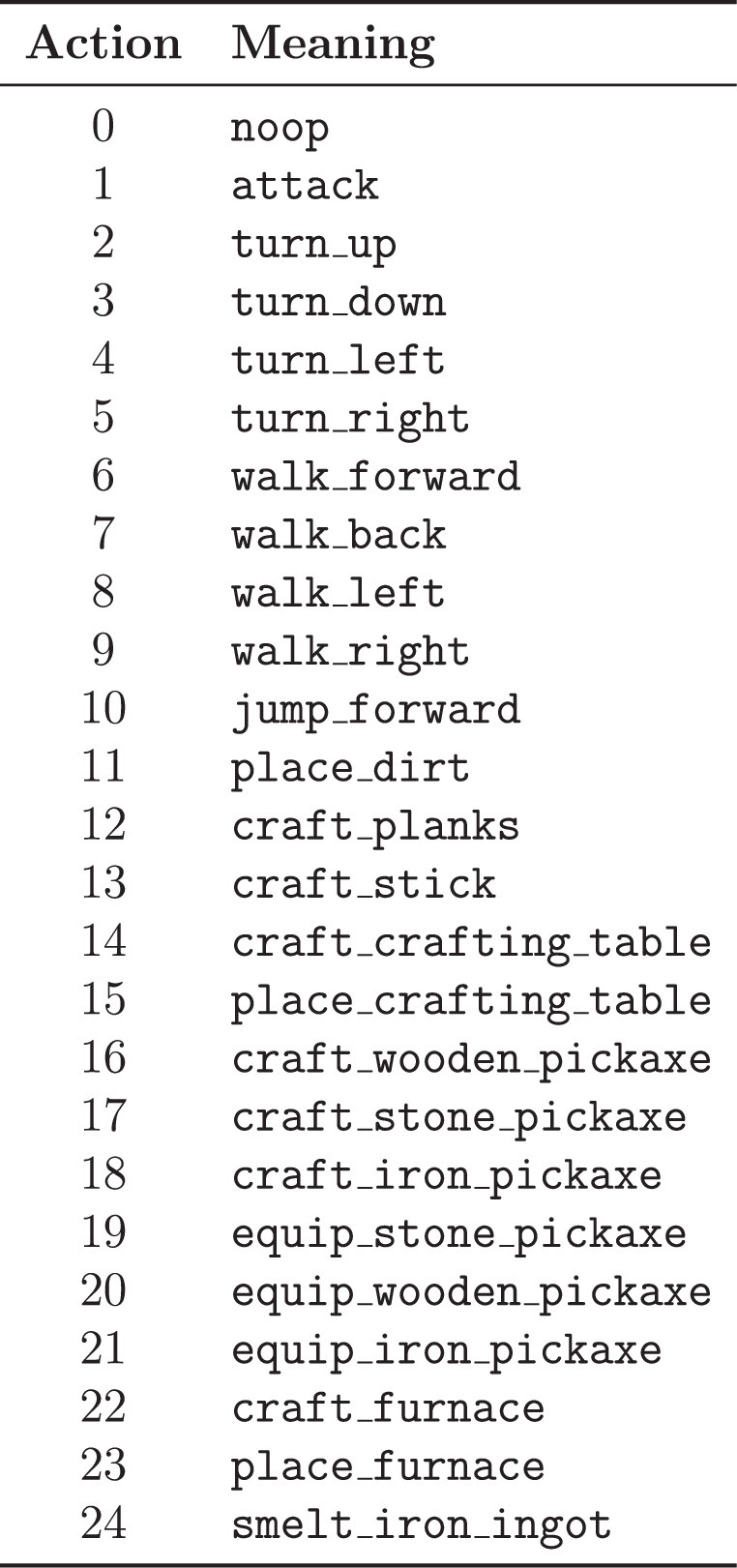
Minecraft categorical action space

## Data Availability

The algorithm generates its own experience data by interacting with the simulated environments at run time, thus no external datasets are used. Datapoints for training curves are available in the code repository.

## Code availability

Source code for Dreamer and all presented experiments is available under an open-source license at https://github.com/danijar/dreamerv3.
