# Supplementary material for: Mastering diverse control tasks through world models
Source: Nature. 2025 Apr 2;640(8059):647–53. doi: 10.1038/s41586-025-08744-2 (PMC12003158; doi:10.1038/s41586-025-08744-2)
Supplement: Supplementary file 1 — Detailed empirical results in the form of learning curve graphs and numeric score tables for all tasks. [file 41586_2025_8744_MOESM1_ESM.pdf]

nature portfolio

https://doi.org/10.1038/s41586-025-08744-2

Supplementary information

Mastering diverse control tasks through world models

In the format provided by the authors and unedited

Atari learning curves

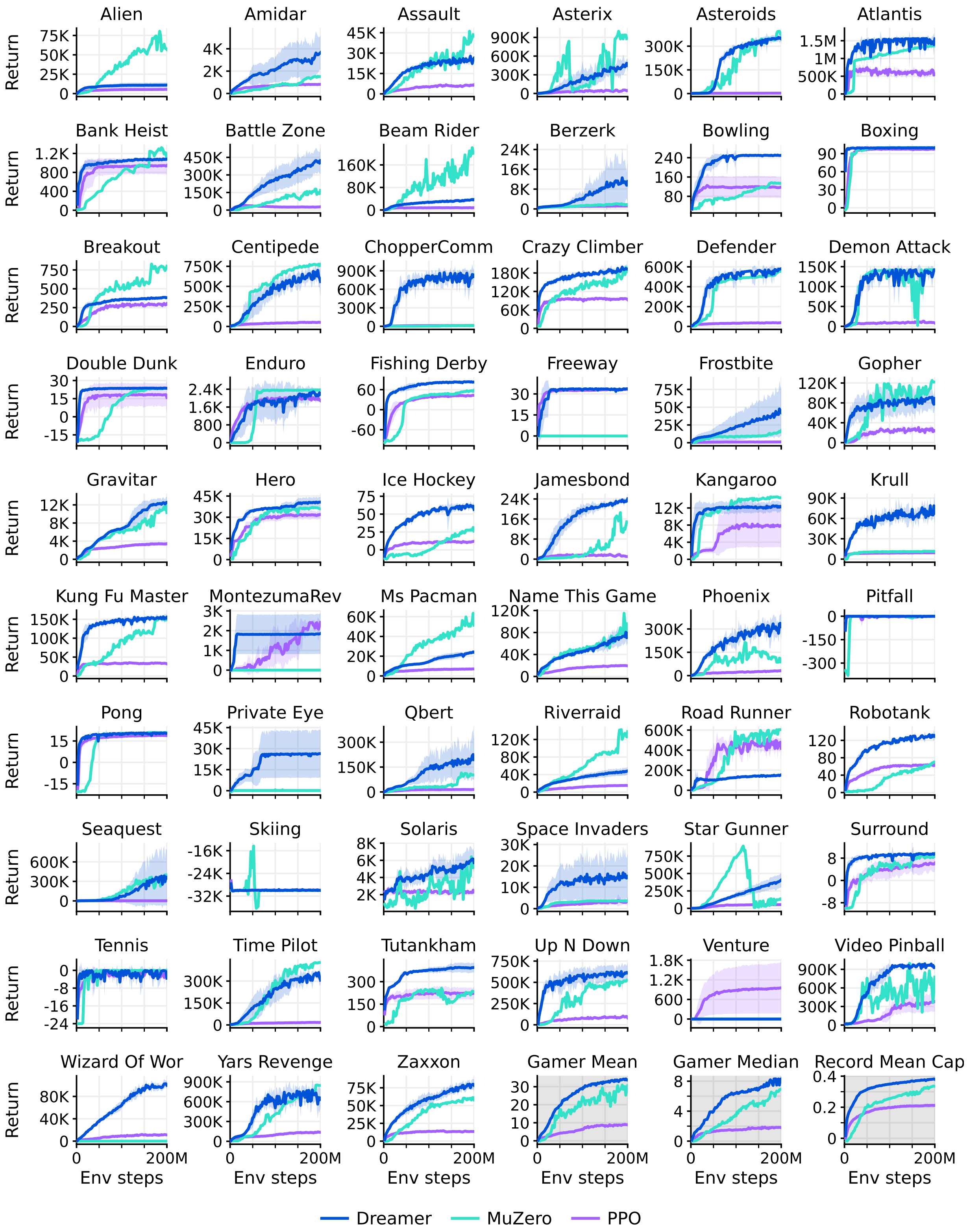

Figure 1: Atari learning curves.

Atari scores

Task	Random	Gamer	Record	PPO	MuZero	Dreamer
Environment steps	—	—	—	200M	200M	200M
Alien	228	7128	251916	5476	**56835**	10977
Amidar	6	1720	104159	817	1517	**3612**
Assault	222	742	8647	6673	**42742**	26010
Asterix	210	8503	1000000	47190	**879375**	441763
Asteroids	719	47389	10506650	2479	**374146**	348684
Atlantis	12850	29028	10604840	539721	1353617	**1553222**
Bank Heist	14	753	82058	946	**1077**	**1083**
Battle Zone	2360	37188	801000	27816	167412	**419653**
Beam Rider	364	16926	999999	7973	**201154**	37073
Berzerk	124	2630	1057940	1186	1698	**10557**
Bowling	23	161	300	118	133	**250**
Boxing	0	12	100	**98**	**100**	**100**
Breakout	2	30	864	299	**799**	384
Centipede	2091	12017	1301709	51833	**774421**	554553
Chopper Command	811	7388	999999	12667	8945	**802698**
Crazy Climber	10780	35829	219900	93176	**184394**	**193204**
Defender	2874	18689	6010500	38270	**554492**	**579875**
Demon Attack	152	1971	1556345	8229	**142509**	**142109**
Double Dunk	–19	–16	22	16	**23**	**24**
Enduro	0	860	9500	1887	**2369**	2166
Fishing Derby	–92	–39	71	43	58	**82**
Freeway	0	30	38	**33**	0	**34**
Frostbite	65	4335	454830	1123	17087	**41888**
Gopher	258	2412	355040	24792	**122025**	87600
Gravitar	173	3351	162850	3436	10301	**12570**
Hero	1027	30826	1000000	31967	36063	**40677**
Ice Hockey	–11	1	36	12	26	**57**
Jamesbond	29	303	45550	1019	14872	**24010**
Kangaroo	52	3035	1424600	7769	**14380**	12229
Krull	1598	2666	104100	9193	11476	**69858**
Kung Fu Master	258	22736	1000000	32335	**148936**	**154893**
Montezuma Revenge	0	4753	1219200	**2368**	0	1852
Ms Pacman	307	6952	290090	7041	**51310**	24079
Name This Game	2292	8049	25220	19441	**85331**	77809
Phoenix	761	7243	4014440	31412	105593	**316606**
Pitfall	–229	6464	114000	–2	**0**	**0**
Pong	–21	15	21	19	**21**	**20**
Private Eye	25	69571	101800	73	100	**26432**
Qbert	164	13455	2400000	14554	102129	**201084**
Riverraid	1338	17118	1000000	14860	**137983**	48080
Road Runner	12	7845	2038100	423995	**604083**	150402
Robotank	2	12	76	63	70	**132**
Seaquest	68	42055	999999	1927	**399764**	356584
Skiing	–17098	–4337	–3272	**–29926**	**–30000**	**–29965**
Solaris	1236	12327	111420	2368	**5860**	**5851**
Space Invaders	148	1669	621535	3489	3639	**15005**
Star Gunner	664	10250	77400	53439	127417	**408961**
Surround	–10	6	6	6	**9**	**9**
Tennis	–24	–8	21	–1	**0**	–3
Time Pilot	3568	5229	65300	17250	**427209**	314947
Tutankham	11	168	5384	225	235	**395**
Up N Down	533	11693	82840	83743	522962	**614065**
Venture	0	1188	38900	**953**	0	0
Video Pinball	16257	17668	89218328	382306	775304	**940631**
Wizard Of Wor	564	4756	395300	10910	0	**99136**
Yars Revenge	3093	54577	15000105	137164	**846061**	675774
Zaxxon	32	9173	83700	13599	58115	**78443**
Gamer median (%)	0	100	3716	180	693	**830**
Gamer mean (%)	0	100	123001	892	3054	**3381**
Record mean (%)	0	13	100	21	66	**74**
Record mean capped (%)	0	13	100	21	34	**38**

Table 1: Atari scores.

ProcGen learning curves

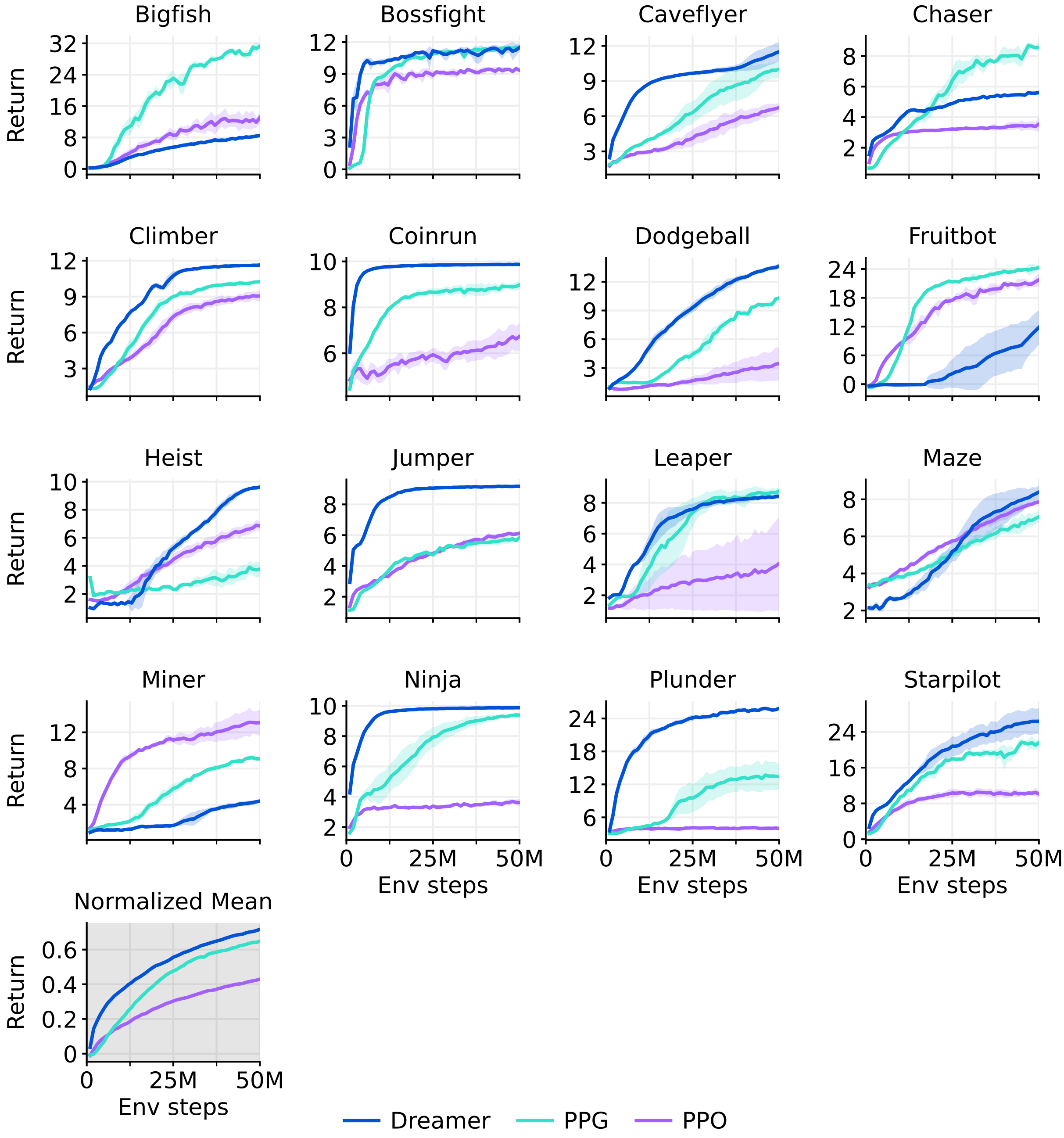

Figure 2: ProcGen learning curves.

ProcGen scores

Task	Original PPO	PPO	PPG	Dreamer
Environment steps	50M	50M	50M	50M
Bigfish	10.9	12.7	**31.3**	8.5
Bossfight	10.5	9.4	**11.5**	**11.5**
Caveflyer	6.0	6.7	10.0	**11.5**
Chaser	4.5	3.5	**8.6**	5.6
Climber	7.6	9.0	10.2	**11.6**
Coinrun	7.9	6.7	9.0	**9.9**
Dodgeball	4.8	3.4	10.3	**13.6**
Fruitbot	20.3	21.7	**24.3**	11.8
Heist	2.2	6.9	3.8	**9.6**
Jumper	5.1	6.1	5.8	**9.2**
Leaper	5.9	4.1	**8.8**	**8.4**
Maze	5.0	7.9	7.1	**8.4**
Miner	7.6	**13.0**	9.1	4.4
Ninja	6.2	3.6	9.4	**9.9**
Plunder	11.2	4.0	13.4	**25.8**
Starpilot	17.0	10.1	21.6	**26.4**
Normalized mean	41.2	42.8	64.9	**71.6**

Table 2: ProcGen scores. The PPO implementation we use under fixed hyperparameters throughout our paper performs on par or better than the original PPO, which its authors describe as highly tuned with near optimal hyperparameters in Cobbe et al. (2021).

DMLab learning curves

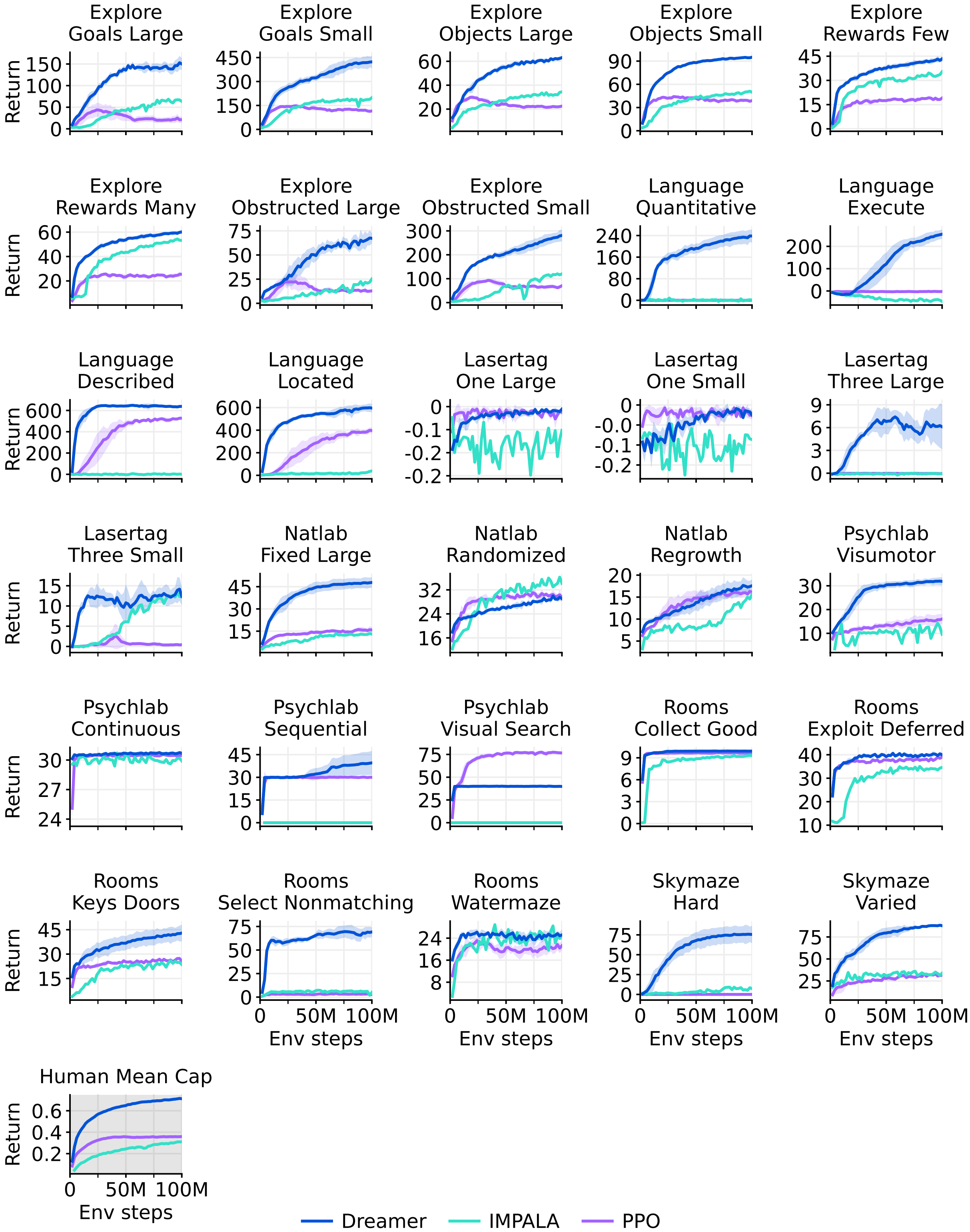

Figure 3: DMLab learning curves.

DMLab scores

Task	R2D2+	IMPALA	IMPALA	IMPALA	PPO	Dreamer
Environment steps	10B	10B	1B	100M	100M	100M
Explore Goal Locations Large	174.7	316.0	137.8	64.2	21.2	**150.5**
Explore Goal Locations Small	460.7	482.0	302.8	196.1	115.1	**423.3**
Explore Object Locations Large	60.6	91.0	55.1	34.3	22.5	**62.9**
Explore Object Locations Small	83.7	100.4	75.9	50.6	38.9	**94.8**
Explore Object Rewards Few	80.7	92.6	46.9	34.6	19.0	**43.1**
Explore Object Rewards Many	75.8	89.4	68.5	53.3	25.5	**60.2**
Explore Obstructed Goals Large	95.5	102.0	57.9	23.7	12.9	**67.2**
Explore Obstructed Goals Small	311.9	372.0	214.9	118.0	70.4	**280.2**
Language Answer Quantitative Question	344.4	362.0	304.7	1.0	0.3	**238.5**
Language Execute Random Task	497.4	465.4	140.8	–44.4	–2.5	**254.7**
Language Select Described Object	617.6	664.0	618.4	0.2	526.9	**637.8**
Language Select Located Object	772.8	731.4	413.0	38.2	397.5	**595.9**
Lasertag One Opponent Large	0.0	0.0	0.0	–0.1	**0.0**	**0.0**
Lasertag One Opponent Small	31.8	0.0	0.0	–0.1	**0.0**	**0.0**
Lasertag Three Opponents Large	28.6	32.2	10.4	–0.1	0.0	**6.2**
Lasertag Three Opponents Small	49.0	57.2	37.1	**12.1**	0.4	**12.5**
Natlab Fixed Large Map	60.6	63.4	53.8	13.0	15.8	**47.7**
Natlab Varying Map Randomized	42.4	47.0	40.5	**35.3**	29.6	29.4
Natlab Varying Map Regrowth	24.6	34.0	25.5	15.3	16.3	**17.5**
Psychlab Arbitrary Visuomotor Mapping	33.1	38.4	16.5	11.9	16.0	**31.9**
Psychlab Continuous Recognition	30.0	28.6	30.0	**30.1**	**30.5**	**30.7**
Psychlab Sequential Comparison	30.0	29.6	0.0	0.0	30.0	**39.5**
Psychlab Visual Search	79.9	80.0	0.0	0.0	**76.6**	39.9
Rooms Collect Good Objects Test	9.9	10.0	9.9	9.3	**9.7**	**9.9**
Rooms Exploit Deferred Effects Test	38.1	62.2	37.6	34.5	**39.0**	**40.0**
Rooms Keys Doors Puzzle	46.2	54.6	36.9	24.2	26.0	**43.1**
Rooms Select Nonmatching Object	63.6	39.0	63.2	4.0	2.7	**69.0**
Rooms Watermaze	49.0	47.0	50.1	23.6	21.2	**25.2**
Skymaze Irreversible Path Hard	76.0	80.0	46.4	7.7	0.0	**75.8**
Skymaze Irreversible Path Varied	76.0	100.0	69.8	32.7	31.3	**87.9**
Human mean capped (%)	85.4	85.1	66.3	31.0	35.9	**71.3**

Table 3: DMLab scores at 100M environment steps and at larger budgets. The IMPALA agent corresponds to "IMPALA (deep)" presented by Kapturowski et al. (2018) who made the learning curves available.

Atari100k learning curves

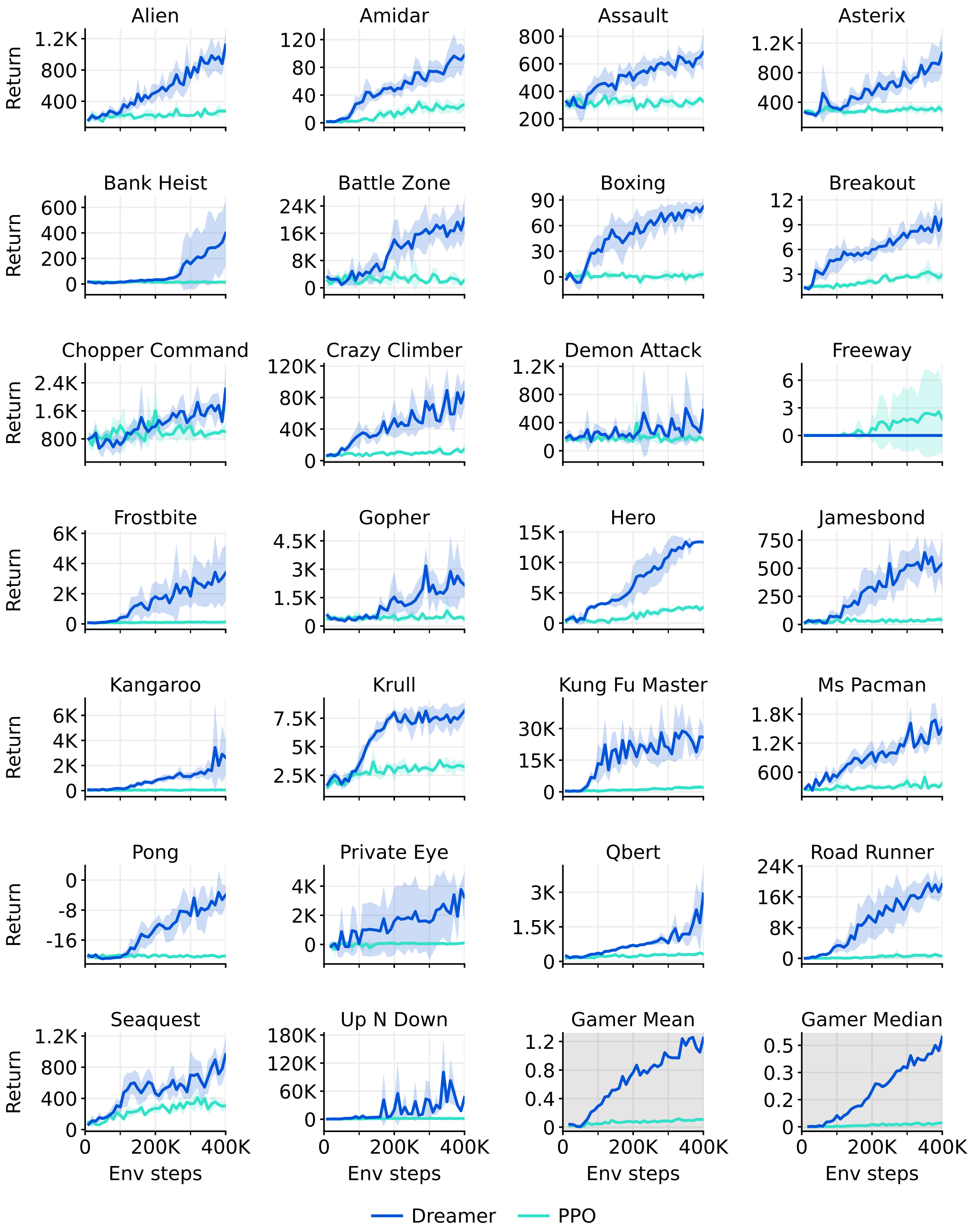

Figure 4: Atari100k learning curves.

Atari100k scores

Task	Random	Human	PPO	SimPLe	SPR	TWM	IRIS	Dreamer
Environment steps	—	—	400K	400K	400K	400K	400K	400K
Alien	228	7128	276	617	842	675	420	**1118**
Amidar	6	1720	26	74	**180**	122	143	97
Assault	222	742	327	527	566	683	**1524**	683
Asterix	210	8503	292	**1128**	962	**1117**	854	1062
Bank Heist	14	753	14	34	345	**467**	53	398
Battle Zone	2360	37188	2233	4031	14834	5068	13074	**20300**
Boxing	0	12	3	8	36	**78**	70	**82**
Breakout	2	30	3	16	20	20	**84**	10
Chopper Command	811	7388	1005	979	946	1697	1565	**2222**
Crazy Climber	10780	35829	14675	62584	36700	71820	59324	**86225**
Demon Attack	152	1971	160	208	518	350	**2034**	577
Freeway	0	30	2	17	19	24	**31**	0
Frostbite	65	4335	127	237	1171	1476	259	**3377**
Gopher	258	2412	368	597	661	1675	**2236**	**2160**
Hero	1027	30826	2596	2657	5859	7254	7037	**13354**
Jamesbond	29	303	41	100	366	362	463	**540**
Kangaroo	52	3035	55	51	**3617**	1240	838	2643
Krull	1598	2666	3222	2205	3682	6349	6616	**8171**
Kung Fu Master	258	22736	2090	14862	14783	24555	21760	**25900**
Ms Pacman	307	6952	366	1480	1318	**1588**	999	**1521**
Pong	–21	15	–20	13	–5	**19**	15	–4
Private Eye	25	69571	100	35	86	87	100	**3238**
Qbert	164	13455	317	1289	866	**3331**	746	2921
Road Runner	12	7845	602	5641	12213	9109	9615	**19230**
Seaquest	68	42055	305	683	558	774	661	**962**
Up N Down	533	11693	1502	3350	10859	15982	3546	**46910**
Gamer mean (%)	0	100	11	33	62	96	105	**125**
Gamer median (%)	0	100	2	13	40	**51**	29	**49**

Table 4: Atari100k scores at 400K environment steps, corresponding to 100k agent steps.

Proprio Control Suite learning curves

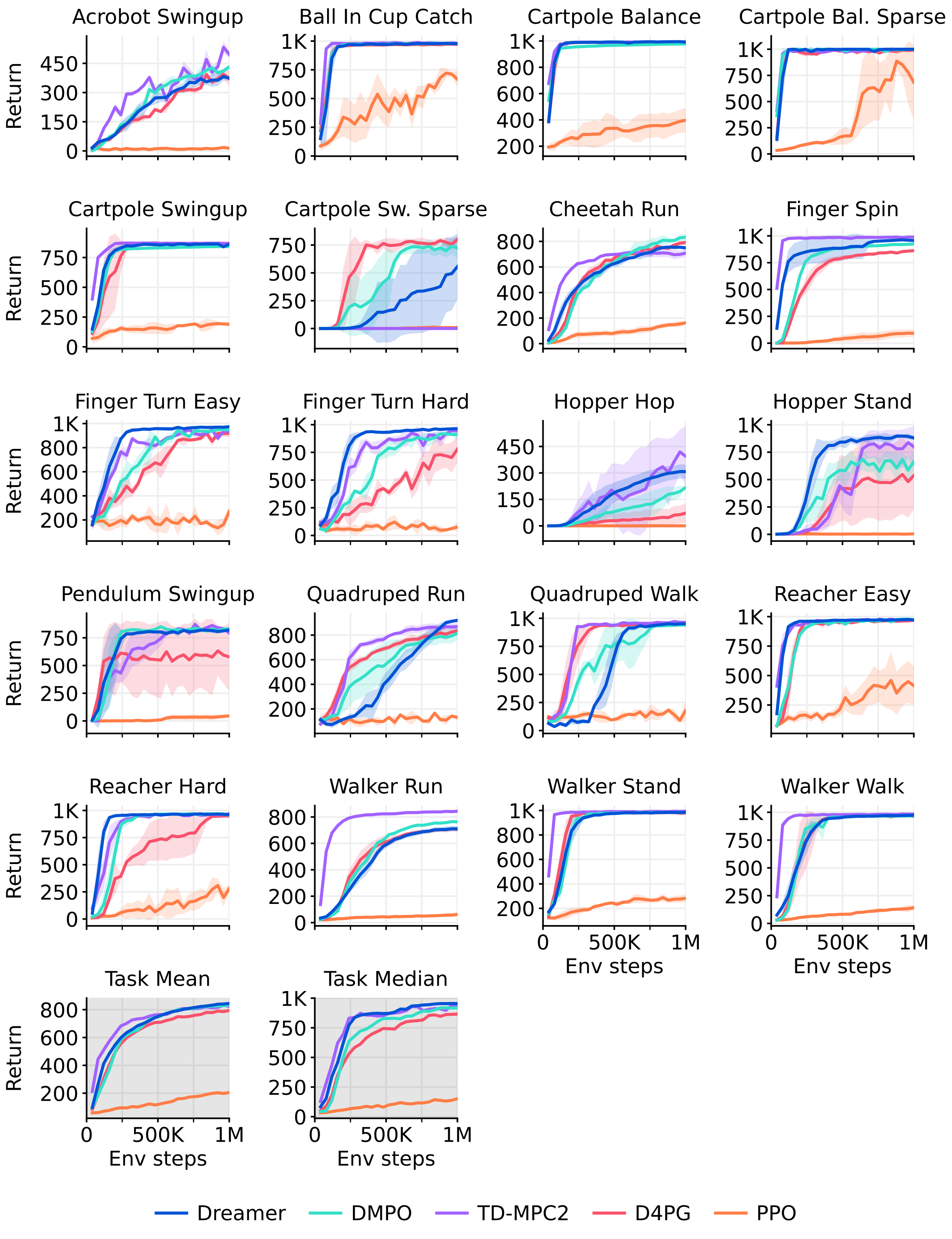

Figure 5: DeepMind Control Suite learning curves under proprioceptive inputs.

Proprio Control Suite scores

Task	PPO	D4PG	TD-MPC2	DMPO	Dreamer
Environment steps	1M	1M	1M	1M	1M
Acrobot Swingup	14	372	**496**	432	378
Ball In Cup Catch	668	**972**	**982**	**979**	**978**
Cartpole Balance	397	**990**	**994**	**977**	**993**
Cartpole Balance Sparse	684	**990**	**1000**	**996**	**1000**
Cartpole Swingup	189	**869**	**864**	**842**	**852**
Cartpole Swingup Sparse	9	**801**	0	722	525
Cheetah Run	163	**791**	708	**832**	752
Finger Spin	92	862	**988**	924	**958**
Finger Turn Easy	270	919	**937**	**950**	**972**
Finger Turn Hard	76	779	**944**	910	**963**
Hopper Hop	0	72	**394**	217	308
Hopper Stand	5	538	799	663	**887**
Pendulum Swingup	45	578	**793**	**832**	**817**
Quadruped Run	131	835	868	816	**916**
Quadruped Walk	178	**953**	**963**	**940**	**951**
Reacher Easy	414	**970**	**967**	**975**	**974**
Reacher Hard	280	**950**	**971**	**956**	**963**
Walker Run	62	707	**843**	763	711
Walker Stand	282	**980**	**994**	**983**	**984**
Walker Walk	141	**963**	**985**	**967**	**973**
Task mean	205	795	**825**	**834**	**843**
Task median	152	865	**941**	**917**	**954**

Table 5: DeepMind Control Suite scores under proprioceptive inputs.

Visual Control Suite learning curves

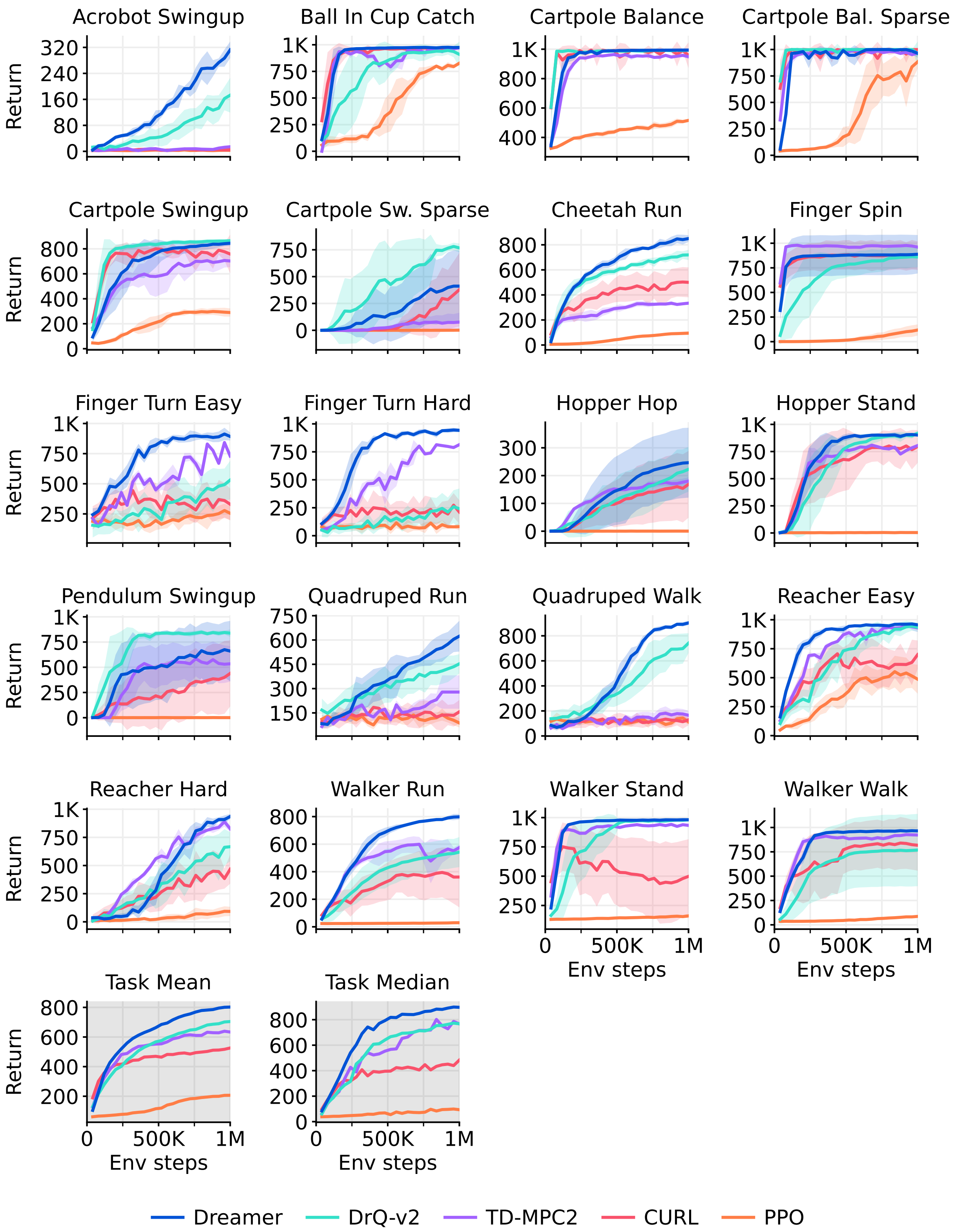

Figure 6: DeepMind Control Suite learning curves under visual inputs.

Visual Control Suite scores

**Task**	**PPO**	**SAC**	**TD-MPC2**	**DrQ-v2**	**Dreamer**
Environment steps	1M	1M	1M	1M	1M
Acrobot Swingup	3	4	14	166	**300**
Ball In Cup Catch	829	176	**971**	920	**975**
Cartpole Balance	516	937	**949**	**992**	**994**
Cartpole Balance Sparse	881	**956**	**968**	**981**	**975**
Cartpole Swingup	290	706	703	**863**	**843**
Cartpole Swingup Sparse	1	149	76	**773**	411
Cheetah Run	95	20	334	716	**846**
Finger Spin	118	291	**961**	861	886
Finger Turn Easy	253	200	730	525	**903**
Finger Turn Hard	79	94	814	247	**944**
Hopper Hop	0	0	180	221	**246**
Hopper Stand	4	5	805	**912**	**904**
Pendulum Swingup	1	592	534	**845**	668
Quadruped Run	88	54	279	450	**614**
Quadruped Walk	112	49	165	726	**897**
Reacher Easy	487	67	**930**	**944**	**960**
Reacher Hard	94	7	827	670	**922**
Walker Run	30	27	575	539	**797**
Walker Stand	161	143	**934**	**979**	**982**
Walker Walk	87	40	**923**	768	**966**
Task mean	206	226	634	705	**802**
Task median	94	81	767	770	**900**

Table 6: DeepMind Control Suite scores under visual inputs.

BSuite performance spectrum

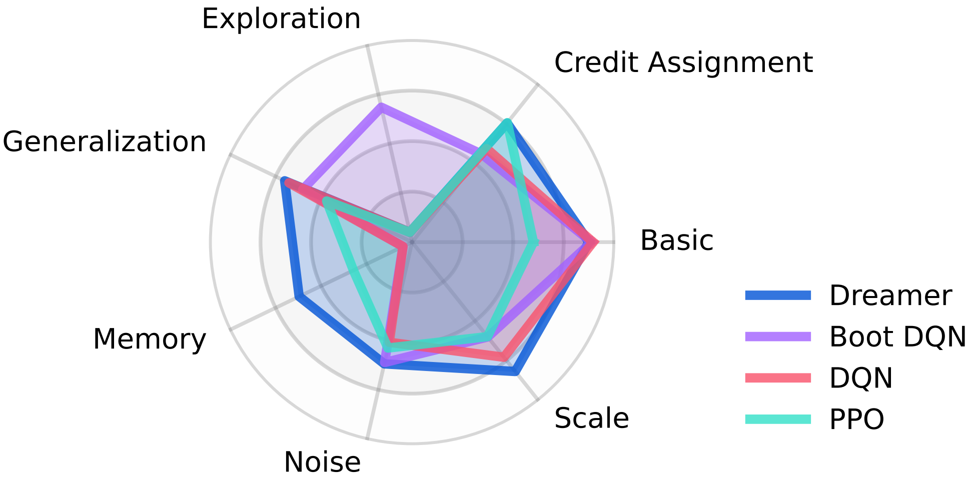

Figure 7: BSuite scores visualized by category. Dreamer exceeds previous methods in the categories scale and memory. The scale category measure robustness to reward scales.

BSuite scores

**Task**	**Random**	**PPO**	**AC-RNN**	**DQN**	**Boot DQN**	**Dreamer**
Bandit	0.00	0.38	**1.00**	0.93	**0.98**	**0.96**
Bandit Noise	0.00	0.61	0.63	0.71	**0.80**	0.75
Bandit Scale	0.00	0.39	0.60	0.74	**0.83**	0.78
Cartpole	0.04	0.84	0.40	0.85	0.69	**0.93**
Cartpole Noise	0.04	0.77	0.20	0.82	0.69	**0.93**
Cartpole Scale	0.04	0.83	0.12	0.72	0.65	**0.92**
Cartpole Swingup	0.00	0.00	0.00	0.00	**0.15**	0.03
Catch	0.00	0.91	0.87	0.92	**0.99**	**0.96**
Catch Noise	0.00	0.54	0.27	0.58	**0.68**	0.53
Catch Scale	0.00	**0.90**	0.17	0.85	0.65	**0.94**
Deep Sea	0.00	0.00	0.00	0.00	**1.00**	0.00
Deep Sea Stochastic	0.00	0.00	0.00	0.00	**0.90**	0.00
Discounting Chain	0.20	0.24	**0.39**	0.25	0.22	**0.40**
Memory Len	0.00	0.17	**0.70**	0.04	0.04	0.65
Memory Size	0.00	0.47	0.29	0.00	0.00	**0.59**
Mnist	0.05	0.77	0.56	**0.85**	**0.85**	0.61
Mnist Noise	0.05	**0.41**	0.22	0.38	0.34	0.34
Mnist Scale	0.05	**0.76**	0.09	0.49	0.31	0.55
Mountain Car	0.10	0.10	0.10	**0.93**	**0.93**	**0.92**
Mountain Car Noise	0.10	0.10	0.10	**0.89**	0.82	**0.87**
Mountain Car Scale	0.10	0.10	0.10	0.85	0.56	**0.90**
Umbrella Distract	0.00	**1.00**	0.09	0.30	0.26	0.74
Umbrella Length	0.00	**0.87**	0.43	0.39	0.39	0.78
Basic	0.04	0.60	0.58	**0.90**	**0.89**	**0.88**
Credit assignment	0.03	**0.76**	0.37	0.59	0.56	**0.75**
Exploration	0.00	0.00	0.00	0.00	**0.68**	0.01
Generalization	0.06	0.47	0.19	**0.68**	0.60	**0.70**
Memory	0.00	0.32	0.49	0.02	0.02	**0.62**
Noise	0.02	0.54	0.24	0.51	**0.61**	**0.62**
Scale	0.04	0.60	0.22	0.73	0.60	**0.82**
Task mean (%)	3	49	32	54	60	**66**
Category mean (%)	3	47	30	49	57	**63**

Table 7: BSuite scores for each task averaged over environment configurations, as well as aggregated performance by category and over all tasks.

Normalization ablations

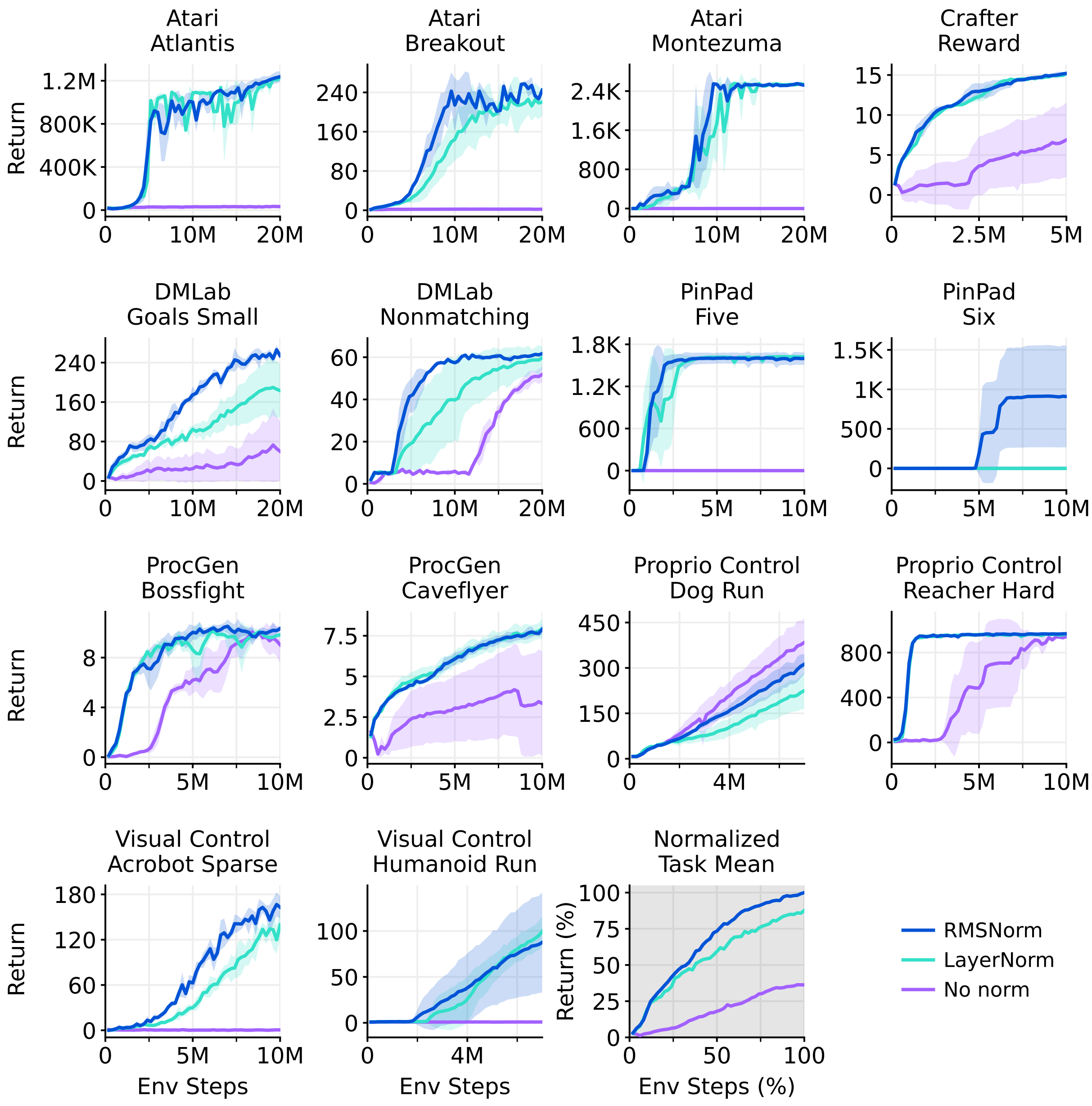

Figure 8: Individual learning curves for the normalization ablation experiment. Both LayerNorm and RMSNorm stabilize the learning dynamics of Dreamer. In practice, we opt for RMSNorm due to its simplicity.

Learning signal ablations

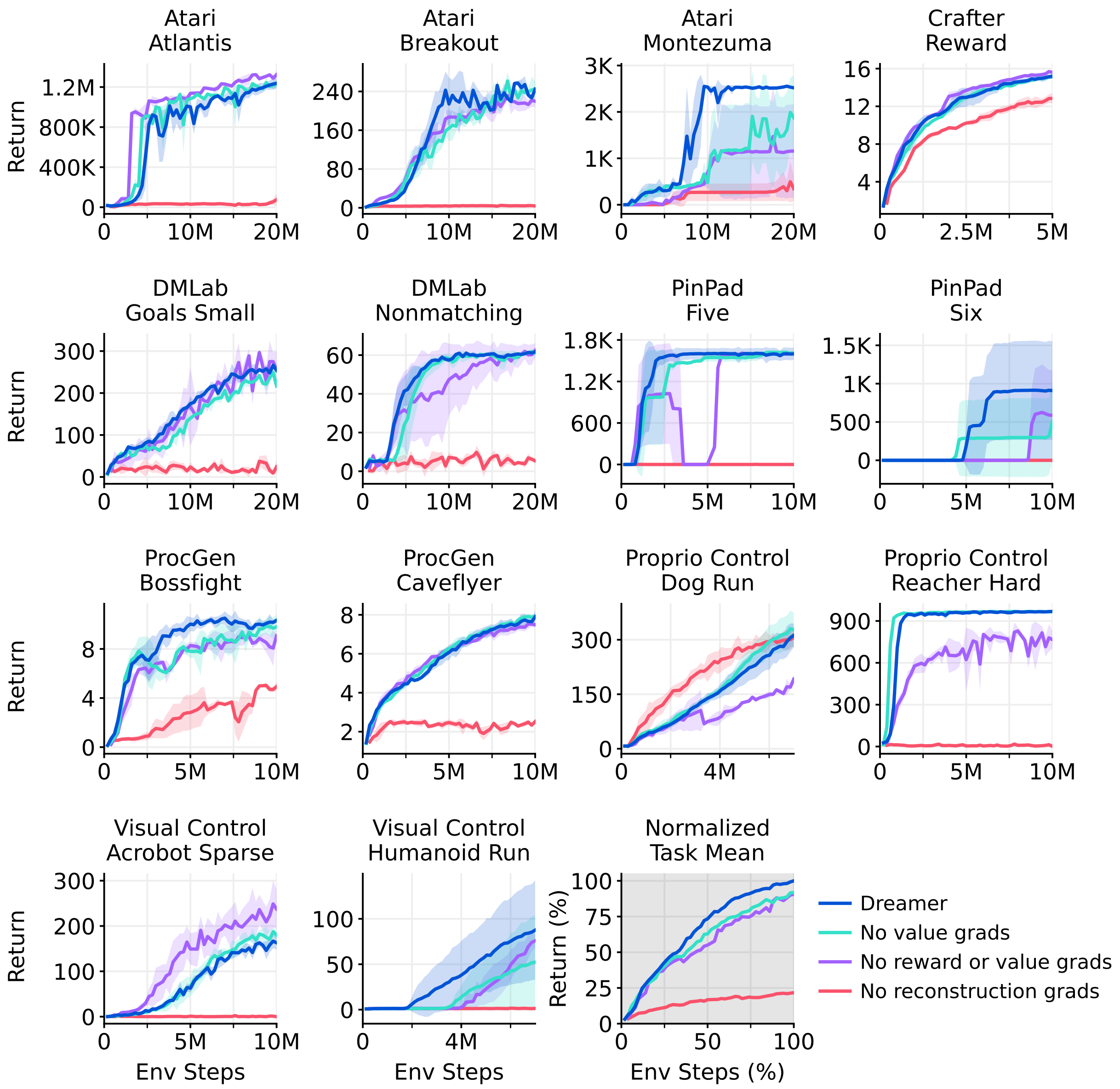

Figure 9: Individual learning curves for the learning signal ablation experiment. Dreamer relies predominantly on the undersupervised reconstruction objective of its world model and additional reward and value gradients further improve performance on a subset of tasks.

Robustness ablations

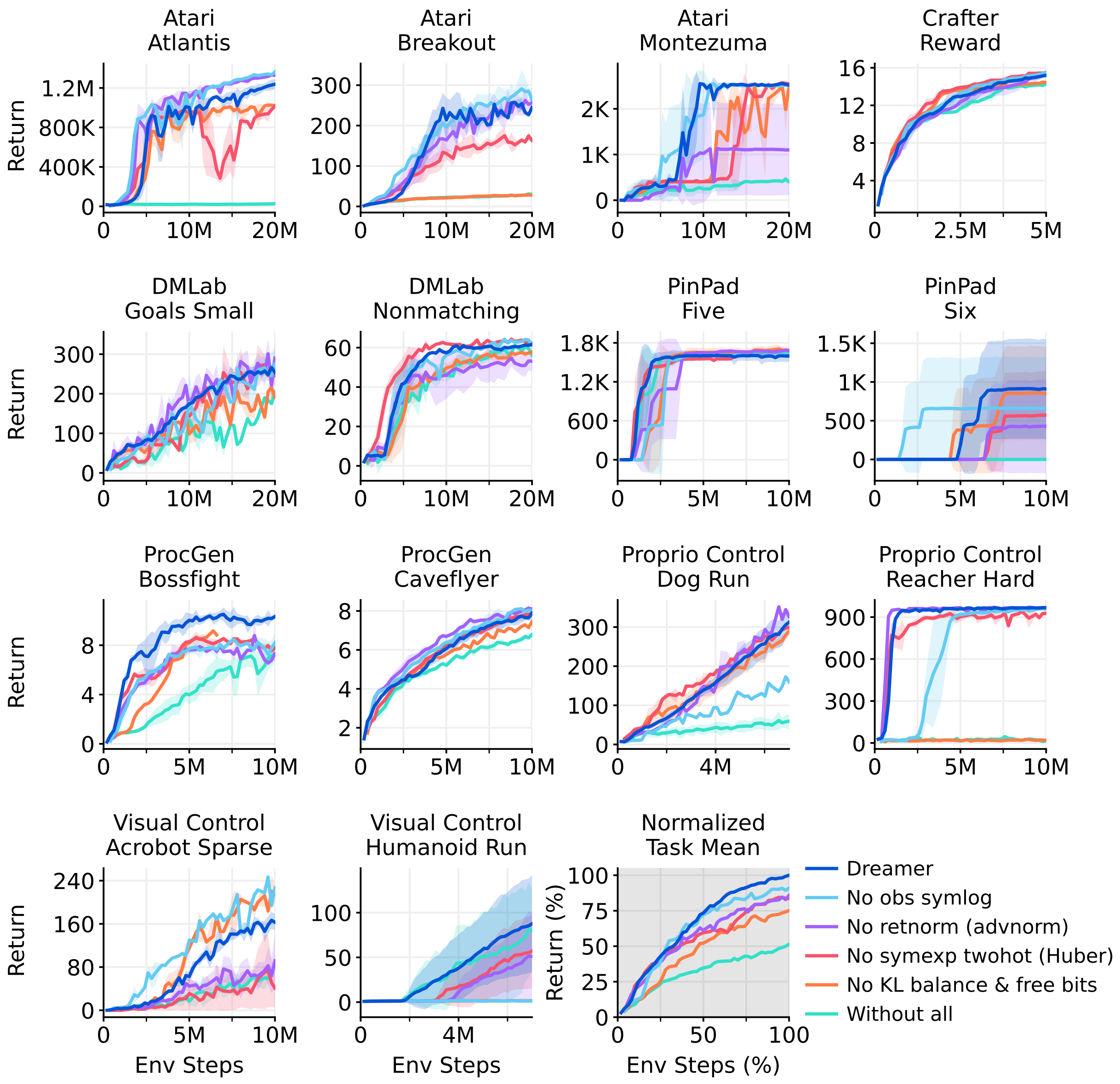

Figure 10: Individual learning curves for the robustness ablation experiment. All robustness techniques contribute to the overall performance of Dreamer, although each individual technique may only improve the performance on a subset of the tasks.
